# A Series of Non-Oxido V^IV^ Complexes of Dibasic ONS Donor Ligands: Solution Stability, Chemical Transformations, Protein Interactions, and Antiproliferative Activity

**DOI:** 10.1021/acs.inorgchem.3c00753

**Published:** 2023-05-08

**Authors:** Atanu Banerjee, Sushree Aradhana Patra, Gurunath Sahu, Giuseppe Sciortino, Federico Pisanu, Eugenio Garribba, M. Fernanda N.N. Carvalho, Isabel Correia, João Costa Pessoa, Hans Reuter, Rupam Dinda

**Affiliations:** † Department of Chemistry, National Institute of Technology, Rourkela 769008, Odisha, India; ‡ Institute of Chemical Research of Catalonia (ICIQ), The Barcelona Institute of Science and Technology, Tarragona 43007, Spain; § Dipartimento di Medicina, Chirurgia e Farmacia, Università di Sassari, Viale San Pietro, Sassari I-07100, Italy; ∥ Centro de Química Estrutural and Departamento de Engenharia Química, Institute of Molecular Sciences, Instituto Superior Técnico, Universidade de Lisboa, Avenida Rovisco Pais, Lisboa 1049-001, Portugal; ⊥ Institute of Chemistry of New Materials, University of Osnabrück, Barbarastraße 6, Osnabruck 49069, Germany

## Abstract

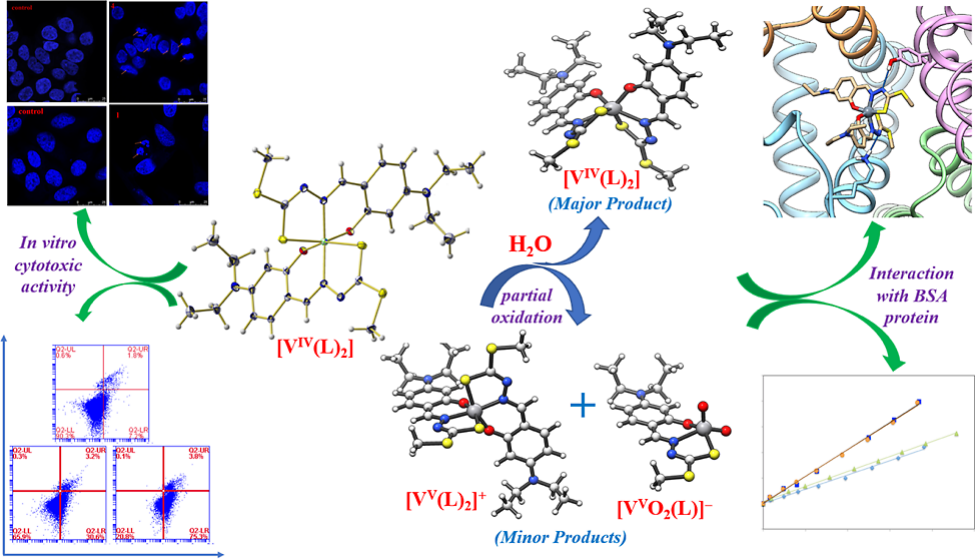

A series of mononuclear non-oxido vanadium(IV) complexes, [V^IV^(L^1–4^)_2_] (**1–4**), featuring tridentate bi-negative ONS chelating S-alkyl/aryl-substituted dithiocarbazate ligands H_2_L^1–4^, are reported. All the synthesized non-oxido V^IV^ compounds are characterized by elemental analysis, spectroscopy (IR, UV–vis, and EPR), ESI-MS, as well as electrochemical techniques (cyclic voltammetry). Single-crystal X-ray diffraction studies of **1**–**3** reveal that the mononuclear non-oxido V^IV^ complexes show distorted octahedral (**1** and **2**) or trigonal prismatic (**3**) arrangement around the non-oxido V^IV^ center. EPR and DFT data indicate the coexistence of *mer* and *fac* isomers in solution, and ESI-MS results suggest a partial oxidation of [V^IV^(L^1–4^)_2_] to [V^V^(L^1–4^)_2_]^+^ and [V^V^O_2_(L^1–4^)]^−^; therefore, all these three complexes are plausible active species. Complexes **1**–**4** interact with bovine serum albumin (BSA) with a moderate binding affinity, and docking calculations reveal non-covalent interactions with different regions of BSA, particularly with Tyr, Lys, Arg, and Thr residues. *In vitro* cytotoxic activity of all complexes is assayed against the HT-29 (colon cancer) and HeLa (cervical cancer) cells and compared with the NIH-3T3 (mouse embryonic fibroblast) normal cell line by MTT assay and DAPI staining. The results suggest that complexes **1**–**4** are cytotoxic in nature and induce cell death in the cancer cell lines by apoptosis and that a mixture of V^IV^, V^V^, and V^V^O_2_ species could be responsible for the biological activity.

## Abstract 2

The synthesis, stability, and chemical transformations in solution, protein interaction, and cytotoxicity of a series of non-oxido [V^IV^(L^1−4^)_2_] complexes of tridentate ONS dithiocarbazate ligands is reported. The results indicate that a mixture of [V^IV^(L^1−4^)_2_], [V^V^(L^1−4^)_2_]^+^, and [V^V^O_2_(L^1−4^)_2_]^−^ species is formed, which may be responsible for the observed activity.

## Introduction

Vanadium is a biologically relevant trace element required by different organisms.^1^ It exists in enzymes such as vanadium-dependent haloperoxidases and nitrogenases^2−5^ and is probably involved in the regulation of phosphate metabolism, thereby playing crucial roles in the human organism.^6−9^ Over the past few years, vanadium compounds have also been demonstrated to have various therapeutic effects,^9−15^ and the correlation between the structure and activity,^16^ reactivity and activity,^17^ as well as the biotransformation of these complexes in blood have been investigated.^18−27^

The interaction of metal complexes with proteins plays crucial roles in pharmaceutical and biomedical applications^4,28−33^ and may affect the action of enzymes and proteins in the organism.^34^ Serum albumins are the major soluble proteins in the blood and have an utmost important role in biological processes; for example, studies have revealed that the *in vivo* circulatory half-life of some metallodrugs was extended as a result of binding with serum albumin.^35,36^

Some oxidovanadium(IV) complexes, particularly [V^IV^O(4,7-Me_2_phen)_2_(SO_4_)] (Metvan, where 4,7-Me_2_phen = 4,7-dimethyl-1,10-phenanthroline), were introduced as promising anticancer drugs for testicular and ovarian cancer,^37^ but they never proceeded to the clinic. There is interest in the study of the pharmaceutical roles of several vanadium complexes,^5,38−46^ such as [bis(cyclopentadienyl)vanadium(IV)] dichloride, [V^IV^O(phen)(H_2_O)_2_]^2+^,^47^ and [V^IV^O(phen)_2_(SO_4_)]^48^ (phen = 1,10-phenanthroline), which exhibited potent antitumor activity against the human nasopharyngeal carcinoma-KB cell line and the clonogenic NALM-6 cell line, respectively. However, more recently, the relevance of addressing the consequences of the probable hydrolysis, ligand exchange, and redox reactions of complexes of labile metal ions in *in vitro* and *in vivo* conditions have been highlighted,^25,49−52^ emphasizing the need to properly disclose which are the relevant biological active species. Namely, incubation media used in studies with mammalian cells also contain appreciable amounts of bovine serum albumin (BSA), which competes for the binding to the metal ions present, often changing the nature of metal-containing species that enter inside cells.^51,52^

Vanadium in its most stable higher oxidation states (+IV and +V) is oxophilic and forms several types of oxido species both in the solid state and in solution, containing strong terminal V=O bonds. It is worth noting that in contrast to those V^IV^/V^V^-oxido species, only very few non-oxido V^IV^ and V^V^ complexes formed by ONS/SS/OS donor ligands have been isolated and structurally characterized.^53−60^ The tendency to form the strong “terminal” V=O bond under most experimental conditions, particularly in water-containing media, makes the formation of non-oxido vanadium(IV) complexes much less probable, particularly under biologically relevant conditions. Even after the discovery of amavadin,^61^ a non-oxido octa-coordinated V^IV^ complex isolated from the mushroom *Amanita muscaria*,^62,63^ studies of their biological activities are still scarce,^43,64−67^ and the determination of the active complex species has been rarely attempted.^16^

Over the last years, the interest in the study of ONS donor ligands has increased significantly due to their ligation properties and potential applications of their transition metal complexes in pharmacology and medicine.^46,68−80^ For example, 3-aminopyridine-2-carboxaldehyde thiosemicarbazone, well known as triapine, is one of the most extensively studied drugs^81^ and has been tested in nearly 30 clinical phases I/II trials as an antiproliferative agent.^82^ Likewise, di-2-pyridylketone-4-cyclohexyl-4-methyl-3-thiosemicarbazone (DpC-TSC) has also entered clinical phase I studies as a chemotherapeutic agent.^83,84^ Out of this class of compounds, similar to thiosemicarbazones, S-substituted dithiocarbazates (DTCs) are also well known for their antitumor, antimicrobial, antifungal, and antibacterial activities.^68,71−76,79,80,85^ Moreover, *N*’-(phenyl-pyridin-2-yl-methylene)-hydrazine carbodithioic acid methyl ester (PHCM) was also found to enhance radiation-induced cell death of an NSCLC cell line, H460, both *in vivo* and *in vitro*. Interestingly, the possible use in bio-imaging and in the treatment of pancreatic cancer of a dithiocarbazate-copper complex has been recently published.^86^

Considering the potential therapeutic use of this type of compounds and our expertise in the field of biological and pharmacological applications of vanadium compounds,^64,65,87−98^ herein we report a new series of non-oxido vanadium(IV) complexes, [V^IV^(L^1–4^)_2_] (**1**–**4**), formed by S-alkyl/aryl substituted dithiocarbazate ligands (H_2_L^1–4^). All these compounds were characterized through elemental analysis, mass spectrometry, spectroscopic techniques, and cyclic voltammetry. Structural evidence for the formation of non-oxido V^IV^ complexes was obtained by the SC-XRD characterization of [V^IV^(L^1–3^)_2_] (**1**–**3**). To the best of our knowledge, this is the second study involving the structural characterization by single-crystal X-ray diffraction analysis (SC-XRD) of non-oxido vanadium(IV) complexes with ONS donor ligands.^53^

Under biological conditions, vanadium complexes often undergo chemical transformations like hydrolysis, redox reactions, ligand-exchange, *and so forth,* which have limited the experimentation of V-based drugs in clinical practice.^3,5,14,41,42,65^ For example, recently, focus has been given to the products formed under biological media by the hydrolysis/oxidation of non-oxido V^IV^-complexes, which have demonstrated to be responsible for the biological properties.^16,43,65,66^ Thus, the possibility of hydrolysis and redox processes of new non-oxido and oxidovanadium(IV) complexes is worth of being explored. Therefore, in this work, we address the chemical transformations, disclosing the solution phase stability of complexes [V^IV^(L^1–4^)_2_] through various physico-chemical techniques. The synthesized complexes **1**–**4** were also tested for their interaction with BSA and *in vitro* antiproliferative activity on HT-29 (colon cancer), HeLa (cervical cancer), and NIH-3T3 (mouse embryonic fibroblast) cell lines.

## Experimental Section

### General Methods and Materials

Most chemicals were obtained from commercial sources. Carbon disulfide (HiMedia), hydrazine hydrate (Loba Chemie), methyl iodide (Sigma-Aldrich), benzyl chloride, and 4-(diethylamino)salicylaldehyde (Sigma-Aldrich) and 2-hydroxy-1-naphthaldehyde (Sigma-Aldrich) were used as received. [V^IV^O(acac)_2_], where acac stands for acetylacetonate, was prepared as described in the literature.^99^ Reagent grade solvents were dried and distilled under nitrogen prior to use according to standard procedures.^100^ HPLC grade DMSO and CH_3_CN were used for spectroscopic and electrochemical studies, whereas EtOH and CH_3_CN were employed for synthesis of ligand precursors^101^ and metal complexes, respectively. BSA for the study of interactions with the compounds was purchased from Sigma-Aldrich. Cell lines (HT-29, HeLa, and NIH-3T3) were obtained from National Centre of Cell Science (NCCS), Pune, India. Dulbecco’s phosphate buffered saline (DPBS), Dulbecco’s modified eagle medium (DMEM), fetal bovine serum (FBS), antibiotic-antimitotic solution, and trypsin EDTA solution were procured from Himedia, Mumbai, India, while 4′,6-diamidino-2-phenylindole dihydrochloride (DAPI) and 3-[4,5-dimethylthiazol-2-yl]-2,5-diphenyltetrazolium (MTT) were purchased from Sigma-Aldrich, India. Elemental analysis measurements were performed on A Vario EL cube CHNS elemental analyzer. A PerkinElmer Spectrum RXI spectrophotometer was used for recording the IR spectra, and ^1^H and ^13^C NMR spectra were recorded on a Bruker Ultrashield 400 MHz spectrometer in the presence of internal standard SiMe_4_. Electronic spectra were obtained on a Lambda25 PerkinElmer spectrophotometer. EPR spectra were recorded at 120 K with an X-band Bruker EMX spectrometer equipped with a HP 53150A microwave frequency counter and a variable temperature unit, the instrumental setting being: microwave frequency 9.40–9.41 GHz; microwave power 20 mW; time constant 81.92 ms; modulation frequency 100 kHz; modulation amplitude 4 Gauss. Positive- (+) and negative-ion (−) mode ESI-MS spectra were recorded with a high-resolution Q Exactive Plus Hybrid Quadrupole-Orbitrap mass spectrometer (Thermo Fisher Scientific) with a flow rate of infusion into the ESI chamber of 5.00 μL/min and a recording range of 50–1000 *m*/*z*. The instrumental conditions for positive-ion mode spectra were: spray voltage 2300 V; capillary temperature 250 °C; sheath gas 5–10 (arbitrary units); auxiliary gas 3 (arbitrary units); sweep gas 0 (arbitrary units); probe heater temperature 50 °C. For negative-ion mode spectra, they were: spray voltage −1900 V; capillary temperature 250 °C; sheath gas 20 (arbitrary units); auxiliary gas 5 (arbitrary units); sweep gas 0 (arbitrary units); probe heater temperature 14 °C. The redox properties were studied under nitrogen by cyclic voltammetry using a three-compartment cell equipped with Pt wire (working and auxiliary) and Ag wire (reference) electrodes, interfaced with VoltaLab PST050 equipment. The cyclic voltammograms of complexes **1**–**4** and free ligands (H_2_L^3–4^) were obtained from electrolyte solutions of NBu_4_BF_4_ in CH_2_Cl_2_ (0.10 M). The potentials were measured in Volt (±10 mV) *vs* saturated calomel electrode (SCE) at 200 mV/s scan rate using [Fe(η^5^-C_5_H_5_)_2_]^0/+^ as an internal reference.

### Synthesis of Ligand Precursors (H_2_L^1–4^)

The dithiocarbazate compounds H_2_L^1–2^ were prepared in a fair yield by the condensation of 4-(diethylamino)salicylaldehyde with the corresponding S-alkyl/aryl substituted dithiocarbazates [S-methyldithiocarbazate (H_2_L^1^) and S-benzyldithiocarbazate (H_2_L^2^)], while H_2_L^3–4^ were synthesized by the reaction of 2-hydroxy-1-naphthaldehyde with the respective dithiocarbazates [S-methyldithiocarbazate (H_2_L^3^) and S-benzyldithiocarbazate (H_2_L^4^)] in a 1:1 ratio in dry ethanol by adopting a reported procedure.^102^ The resulting yellowish compounds were filtered and then washed with ethanol and finally dried over fused CaCl_2_ in a desiccator.

#### H_2_L^1^

Yield: 74%. Anal. Calcd for C_13_H_19_N_3_OS_2_: C, 52.49; H, 6.44; N, 14.13; S, 21.56. Found: C, 52.28; H, 6.72; N, 14.48; S, 21.23. IR (KBr pellet, cm^–1^): 3642 ν(O–H); 3189 ν(N–H); 1630 ν(C=N); 1494 ν(C–O/phenolate); 1317 ν(C=S). ^1^H NMR (400 MHz, DMSO-*d*_6_): δ (ppm) 13.13 (s, 1H, NH), 10.13 (s, 1H, OH), 8.31 (s, 1H, HC=N−), 7.31–6.12 (m, 3H, Aromatic), 3.31 (m, 4H, −CH_2_–N), 2.51 (s, 3H, CH_3_–S), 1.11 (m, 6H, CH_3_). ^13^C NMR (100 MHz, DMSO-*d*_6_): δ (ppm) 194.48, 159.79, 151.31, 147.81, 131.13, 106.51, 104.86, 97.66, 44.35, 17.12, 12.98.

#### H_2_L^2^

Yield: 82%. Anal. Calcd C_19_H_23_N_3_OS_2_: C, 61.09; H, 6.21; N, 11.25; S, 17.17. Found: C, 61.28; H, 6.28; N, 11.41; S, 17.49. IR (KBr pellet, cm^–1^): 3683 ν(O–H); 3109 ν(N–H); 1624 ν(C=N); 1516 ν(C–O/phenolate); 1340 ν(C=S). ^1^H NMR (400 MHz, DMSO-*d*_6_): δ (ppm) 13.18 (s, 1H, NH), 10.09 (s, 1H, OH), 8.31 (s, 1H, HC=N−), 7.42–6.07 (m, 8H, Aromatic), 4.49 (s, 2H, CH_2_–S), 3.36 (m, 4H, CH_2_–N), 1.08 (m, 6H, CH_3_). ^13^C NMR (100 MHz, DMSO-*d*_6_): δ (ppm) 192.70, 159.76, 151.36, 147.74, 137.45, 130.89, 129.64, 129.53, 129.45, 106.46, 104.87, 97.54, 44.35, 37.85, 12.95.

#### H_2_L^3^

Yield: 76%. Anal. Calcd C_13_H_12_N_2_OS_2_: C, 56.49; H, 4.38; N, 10.14; S, 23.20. Found: C, 56.53; H, 4.40; N, 10.20 S, 23.18. IR (KBr pellet, cm^–1^): 3445 ν(O–H); 3156 ν(N–H); 1620 ν(C=N); 1577 ν(C–O/phenolate); 1325 ν(C=S). ^1^H NMR (400 MHz, 157 DMSO-*d*_6_): δ (ppm) 13.42 (s, 1H, NH), 11.08 (s, 1H, OH), 9.19 (s, 1H, HC=N−), 8.80–7.23 (m, 6H, Aromatic), 2.50 (s, 3H, CH_3_–S). ^13^C NMR (100 MHz, DMSO- *d*_6_): δ (ppm) 196.72, 158.69, 146.23, 134.21, 131.72, 129.36, 128.71, 128.61, 124.18, 123.73, 118.70, 109.83, 17.38.

#### H_2_L^4^

Yield: 80%. Anal. Calcd C_19_H_16_N_2_OS_2_: C, 64.74; H, 4.58; N, 7.95; S, 18.19. Found: C, 64.80; H, 4.55; N, 7.96; S, 18.23. IR (KBr pellet, cm^–1^): 3458 ν(O–H); 3157 ν(N–H); 1622 ν(C=N); 1572 ν(C–O/phenolate); 1327 ν(C=S). ^1^H NMR (400 MHz, DMSO-*d*_6_): δ (ppm) 13.46 (s, 1H, NH), 11.09 (s, 1H, OH), 9.20 (s, 1H, HC=N−), 8.71–7.21 (m, 11H, Aromatic), 4.59 (s, 2H, CH_2_–S). ^13^C NMR (100 MHz, DMSO-*d*_6_): δ (ppm) 194.73, 157.25, 145.20, 136.09, 132.66, 131.72, 130.55, 127.34, 127.20, 126.80, 125.74, 124.60, 123.69, 120.19, 120.71, 117.25, 116.11, 112.89, 45.15.

### Synthesis of Complexes [V^IV^(L^1–4^)_2_] (1–4)

The non-oxido vanadium(IV) compounds, formulated as [V^IV^(L^1–4^)_2_], were synthesized by mixing the ONS donor dithiocarbazates H_2_L^1–4^ with [V^IV^O(acac)_2_] in stoichiometric amounts (2:1 molar ratio) in CH_3_CN and allowing it to reflux for 3 h. Greenish black-colored crystalline compounds (**1–4**) were directly obtained from the reaction mixtures. They were then collected by filtration, washed properly with hexane, and dried over CaCl_2_. Suitable crystals of monomeric **1–3** compounds for single-crystal X-ray measurements were collected from their respective reaction pots containing CH_3_CN solvent.

#### [V^IV^(L^1^)_2_] (**1**)

Yield: 70%. Anal. Calcd for C_26_H_34_N_6_O_2_S_4_V: C, 48.66; H, 5.34; N, 13.09; S, 19.98. Found: C, 48.61; H, 5.33; N, 13.15; S, 19.99. IR (KBr pellet, cm^–1^): 1612 ν(C=N). UV–vis (DMSO) [λ_max_, nm (ε, M^–1^ cm^–1^)]: 629 (472), 529 (659), 390 (2157). ESI-MS (CH_3_CN): *m*/*z* 664.0960 [M + Na]^+^.

#### [V^IV^(L^2^)_2_] (**2**)

Yield: 76%. Anal. Calcd for C_38_H_42_N_6_O_2_S_4_V: C, 57.48; H, 5.33; N, 10.58; S, 16.15. Found: C, 57.51; H, 5.34; N, 10.63; S, 16.13. IR (KBr pellet, cm^–1^): 1613 ν(C=N). UV–vis (DMSO) [λ_max_, nm (ε, M^–1^ cm^–1^)]: 628 (450), 526 (730), 401 (2046). ESI-MS (CH_3_CN): *m*/*z* 816.1585 [M + Na]^+^.

#### [V^IV^(L^3^)_2_] (**3**)

Yield: 72%. Anal. Calcd for C_26_H_20_N_4_O_2_S_4_V: C, 52.08; H, 3.36; N, 9.34; S, 21.39. Found: C, 52.09; H, 3.33; N, 9.29; S, 21.40. IR (KBr pellet, cm^–1^): 1592 ν(C=N). UV–vis (DMSO) [λ_max_, nm (ε, M^–1^ cm^–1^)]: 653 (397), 522 (472), 382 (1869), 333 (2487). ESI-MS (CH_3_CN): *m*/*z* 621.9814 [M + Na]^+^.

#### [V^IV^(L^4^)_2_] (**4**)

Yield: 74%. Anal. Calcd for C_38_H_28_N_4_O_2_S_4_V: C, 60.70; H, 3.75; N, 7.45; S, 17.06. Found: C, 60.73; H, 3.71; N, 7.49; S, 17.09. IR (KBr pellet, cm^–1^): 1602 ν(C=N). UV–vis (DMSO) [λ_max_, nm (ε, M^–1^ cm ^–1^)]: 644 (401), 528 (611), 385 (1932), 334 (2531). ESI-MS (CH_3_CN): *m*/*z* 774.0427 [M + Na]^+^.

### X-ray Crystallography

Crystal and intensity data on complexes **1**–**3** were collected on a standard Bruker Kappa APEX II CCD-based 4-circle X-ray diffractometer using graphite-monochromated Mo-K_α_ radiation. Data were corrected for Lorentz and polarization effects; no extinction corrections were applied, but absorption correction was taken into account on a semi-empirical basis using equivalent scans with *R*_int_ = Σ|F_o_^2^ – F_o_^2^(mean)|/Σ[F_o_^2^] and *R*_σ_ = Σ[σ(F_o_^2^)]/Σ[F_o_^2^].

The structures were solved by direct methods (SHELXS^103^) and refined by full-matrix least squares techniques against F_o_^2^ (SHELXL-97 and SHELXL-2014). Atomic scattering factors were taken from International Tables for Crystallography.^104^ The final agreement indices are *R*_1_ = Σ||*F*_o_| – |*F*_c_||/Σ|*F*_o_| and *wR*_2_ = {Σ[*w*(*F*_o_^2^ – *F*_c_^2^)^2^]/Σ[(*wF*_o_^2^)^2^]}^1/2^, and weighting function used is *w* = 1/[σ^2^(*F*_o_^2^) + (*aP*)^2^ + *bP*] with *P* = (max*F*_o_^2^ + 2*F*_c_^2^)/3. Goof = *S* = {Σ[*w*(*F*_o_^2^ – *F*_c_^2^)^2^]/(*n*–*p*)}^1/2^, where *n* is the number of reflections and *p* is the total number of parameters refined. Although the hydrogen atoms could be localized in difference Fourier syntheses, those of the organic moieties were refined in geometrically optimized positions riding on the corresponding carbon atoms, with C–H distances at *T* = 293 K/100 K of 0.96/0.98 Å (−CH_3_), 0.97/0.99 Å (−CH_2_−), 0.93/0.95 Å (−CHsp^2^), and common isotropic thermal displacement parameters for chemical equivalent H-atoms. Figures were drawn using DIAMOND^105^ and Mercury.^106^ In the ball-and-stick models, all atoms are drawn as thermal displacement ellipsoids of the 50% level with exception of the hydrogen atoms, which are shown as spheres of arbitrary radii. Hydrogen bonds are drawn in red as dashed sticks. Crystallographic data as well as structure solution and refinement details are summarized in Table S1. XP (SIEMENS Analytical X-ray Instruments, Inc.1994) was used for structure representations.

### Fluorescence Competition Titrations

The interaction between complexes **1**–**4** and BSA was studied using fluorescence quenching spectroscopy on a PerkinElmer LS 55 instrument. The instrumental response was corrected by means of a correction function provided by the manufacturer. The fluorescence experiments were carried out at room temperature and are steady-state measurements. Electronic absorption spectra were measured on a PerkinElmer Lambda35.

Fatty acid free BSA stock solution was prepared in PBS buffer (10 mM, pH 7.4), and the concentration was determined spectrophotometrically considering the extinction coefficient 44,309 M^–1^ cm^–1^ at 280 nm.^107^ Stock solutions of the complexes (*ca.* 8 × 10^–4^ M) were prepared in DMSO and further diluted (1:10) immediately before running the experiments. Aliquots of each complex (*ca.* 80 μM in DMSO) were added directly to a cuvette containing the BSA solution (*ca.* 1–2 μM) in PBS buffer to obtain complex/BSA molar ratios ranging from 0.1 to 3.5. After addition of the complex, the solutions were allowed to stand for 5 min before measurements. Fluorescence emission spectra were measured at room temperature with λ_ex_ = 295 nm between 310 and 500 nm. Fluorescence spectra containing the same amount of complex but no BSA were recorded and subtracted from each corresponding emission spectrum of the mixtures. The DMSO amount in the titrations was always lower than 5% (v/v). UV–vis spectra were measured for each solution, and the absorbance at excitation and emission wavelengths were used to make corrections for inner filter effects and reabsorption.^108,109^

The Stern–Volmer equation was used to fit the fluorescence quenching data^109^
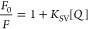
1where *F*_0_ and *F* are the fluorescence intensities of BSA in the absence and presence of quencher, respectively, [*Q*] is the quencher concentration (the vanadium complex in our systems), and *K*_SV_ is the Stern–Volmer quenching constant, obtained from the slope of the *vs* [*Q*] plot.

The binding constants (*K*_BC_) were estimated through linearization of the quenching data, considering the equilibrium between free and bound quencher molecules when they bind independently to *n* equivalent sites of BSA
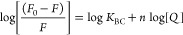
2

### Computational Studies

#### DFT Calculations

The geometry of non-oxido vanadium(IV) [V^IV^(L^1–4^)_2_], non-oxido vanadium(V) [V^V^(L^1–4^)_2_]^+^, and dioxidovanadium(V) [V^V^O_2_(L^1–4^)]^−^ complexes was optimized with Gaussian 16, rev. B01,^110^ at the DFT theory level using the hybrid B3P86 functional combined with the split-valence 6-311g basis set. This method has been successfully applied and discussed in the literature for the geometry prediction of vanadium species.^111,112^ For all the structures, minima were verified through frequency calculations. The ^51^V hyperfine coupling tensor ***A*** of all [V^IV^(L^1–4^)_2_] complexes was predicted with ORCA software version 4.0^115^ using the B2PLYP functional and 6-311g(d,p) basis set, as suggested previously.^114^ The percent deviation (PD) of the absolute calculated value (|*A*_z_|^calcd^) from the absolute experimental value (|*A*_z_|^exptl^) was obtained as 100 × [(|*A*_z_|^calcd^ – |*A*_z_|^exptl^)/|*A*_z_|^exptl^]. UV–vis vertical excitations were simulated on the time-dependent density functional theory (TD-DFT) framework. The simulations were carried out on the previously optimized geometries using the range separated CAM-B3LYP functional and the 6-31+g(d) basis set, according to the method proposed in the literature.^53^ The predicted electronic spectra were generated using Gabedit software,^115^ and the molecular orbitals (MOs) involved in the transitions were simulated performing a Mulliken population analysis (MPA) with Gaussian 16 at the same level of theory used for the TD-DFT calculations and identified with the AOMix package (vers. 6.52).^116^

#### Molecular Docking on BSA

Docking assays toward BSA were carried out with GOLD 5.8 software,^117^ according to the procedures recently reported.^118−122^ The calculations were based on the XRD structure of BSA (PDB code: 4F5S),^123^ removing all the crystallographic waters and adding hydrogen atoms with the UCSF Chimera program.^124^ Non-covalent classical dockings, in which the complexes [V^IV^(L^1–4^)_2_]/[V^V^(L^1–4^)_2_]^+^ and [V^V^O_2_(L^1–4^)]^−^ can interact only through second-coordination sphere interactions, were carried out. The blind exploration was performed on the rigid protein, building four evaluation spheres of 20 Å containing globally the whole protein. Genetic algorithm (GA) parameters have been set in 50 GA runs and a minimum of 100,000 operations. The other parameters of GA were set to default. The scoring (*Fitness* of GoldScore) was determined through the recent validated versions of GoldScore accounting for vanadium-complexes surface interactions.^121^ The best solutions (binding poses), were evaluated taking into account the mean (*F*_mean_) and the highest value (*F*_max_) of the scoring and population of the cluster containing the best pose.

### Cytotoxicity Experiments

#### MTT Assay

HT-29 (colon cancer), HeLa (cervical cancer), and NIH-3T3 (mouse embryonic fibroblasts) cells were maintained in DMEM supplemented with 10% FBS, penicillin–streptomycin solution in a humidified (95% humidity) CO_2_ incubator (5% CO_2_). Thereafter, cells were harvested from the logarithmic phase and seeded into a 96-well plate at a concentration of 8 × 10^4^ cells per well. After overnight seeding, cells were treated with different concentrations (2.5, 5, 10, 20, and 50 μM) of the tested ligands and vanadium complexes (H_2_L^1–4^ and **1**–**4**) for 48 h. The compounds’ stock solutions were prepared in DMSO. DMEM was used to prepare the final working concentration of the compounds, so that the final amount of DMSO is less than 2%. The effect of the complexes on the viability of the cells was evaluated through MTT dye reduction assay by measuring the optical density at 595 nm.^93^ DMSO alone, used to dissolve the complexes, was also employed for the control group treatment.

#### Nuclear Staining Assay

Morphological changes of the nucleus and its structural integrity during cell death were analyzed by the DAPI staining method.^91,93^ Briefly, the cells (HeLa and HT-29) were treated with the tested compounds **1–4** at a concentration of 10 μM and were kept for 24 h. After this, fixation of the cells was done, followed by washing with PBS. Subsequently, the cells were stained with DAPI and kept in dark for 10 min. Finally, the cells were washed twice with PBS and taken for imaging in a Leica TCS SP8 confocal laser scanning microscope with 25× magnification.

#### Apoptosis Assay

The apoptotic population induced by complexes **1** and **2** in HT-29 cells was evaluated using Annexin V-FITC Apoptosis Detection Kit (Abgenex) by a flow cytometer (BD Accuri C6 flow cytometer; BectoneDickinson).^125^ HT-29 cells (3 × 10^5^ cells/well) were cultured in a 6-well plate for 24 h. After the cells were adhered, different concentrations (5 and 10 μM) of the tested complexes were treated and incubated for further 48 h. Subsequently, the cells were collected by trypsinization and then suspended in 1X binding buffer. Finally, the suspension was stained with 5 μL of Annexin V and PI at room temperature and incubated for 15 min in the dark. Then, the samples were immediately analyzed under a BD Accuri C6 flow cytometer.

## Results and Discussion

### Synthesis

**Scheme 1:**
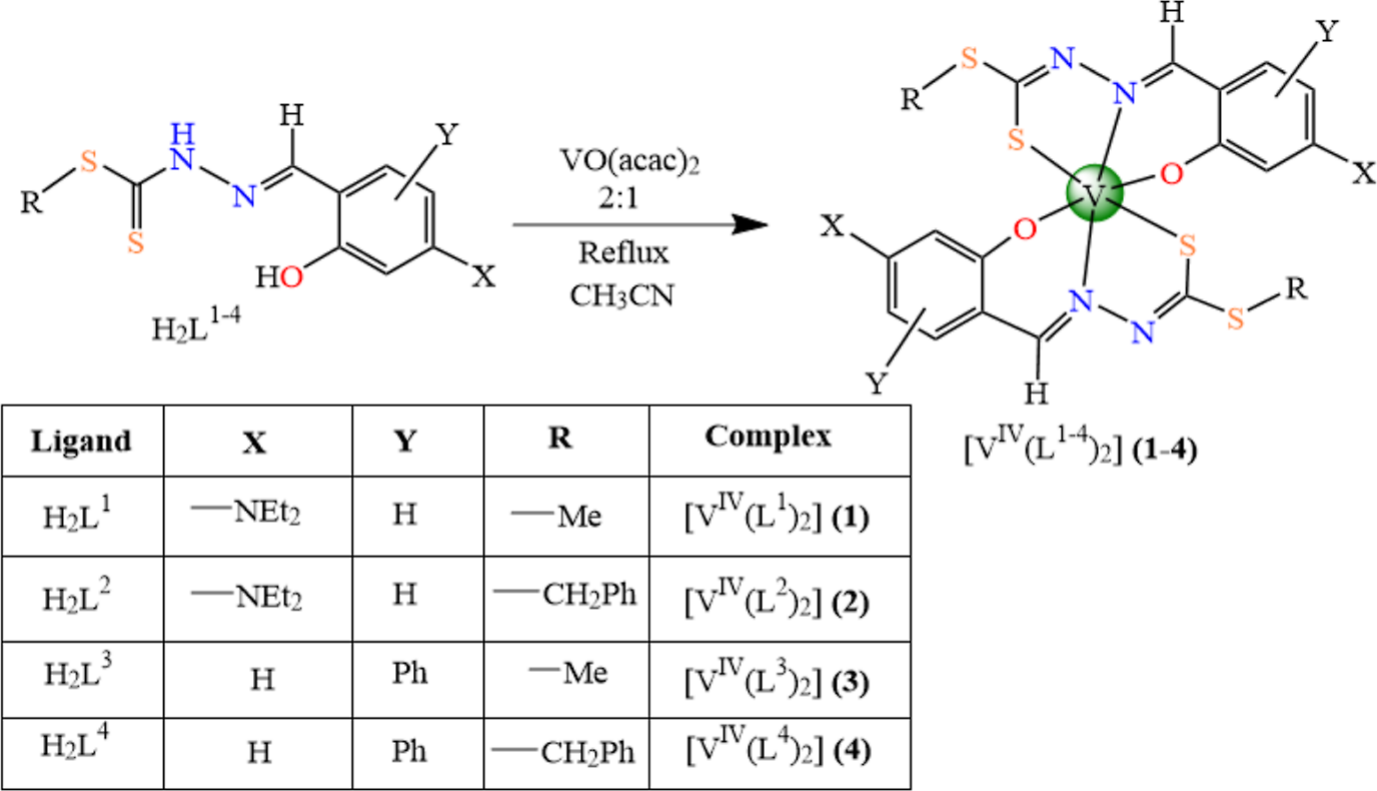
Schematic Representation of the Synthesis of Complexes [V^IV^(L^1–4^)_2_] (**1–4**)

The non-oxido vanadium(IV) complexes (**1–4**) were synthesized by the reaction of tridentate dibasic ONS donor dithiocarbazates H_2_L^1–4^ with [V^IV^O(acac)_2_] in a 2:1 molar ratio in CH_3_CN medium (Scheme 1). All compounds are stable in the solid state and do not oxidize under air. They are soluble in polar aprotic solvents like DMSO, DMF, DCM, THF, and CH_3_CN and are partially soluble in aqueous media. The solid-state characterization of all compounds was done through elemental analysis, FTIR, and also with single-crystal X-ray diffraction studies of **1–3**, while the solution phase characterization was carried out through UV–vis, ESI-MS spectrometry, and EPR spectroscopy. Details of all the characterizations are described in the following sections.

### IR Spectroscopy

The spectral data of compounds **1**–**4** are summarized in the Experimental Section. Some of the IR bands of the complexes show a shift in wavenumbers, compared to those of their respective dithiocarbazate precursors (H_2_L^1–4^). A band in the region 3683–3445 cm^–1^ can be assigned to the stretching vibration of the aromatic O–H of the free ligands, which is absent in the corresponding metal complexes due to the deprotonation and coordination. The spectra exhibit the characteristic bands of the dithiocarbazate ligands, which include a strong signature band at ∼1630–1620 cm^–1^ of the ν(C=N) vibration,^126^ together with a strong band in the region 1577–1494 cm^–1^ due to the C–O phenolic group.^53^ These bands shift toward lower frequency upon coordination to the metal center. The absence of the strong characteristic ν(V=O) stretching band in the region 900–1000 cm^–1^ indicates the formation of a non-oxido vanadium center in **1**–**4**.^53,64,65,127^

### Single-Crystal X-ray Diffraction Analysis of Complexes **1**–**3**

**Figure 1:**
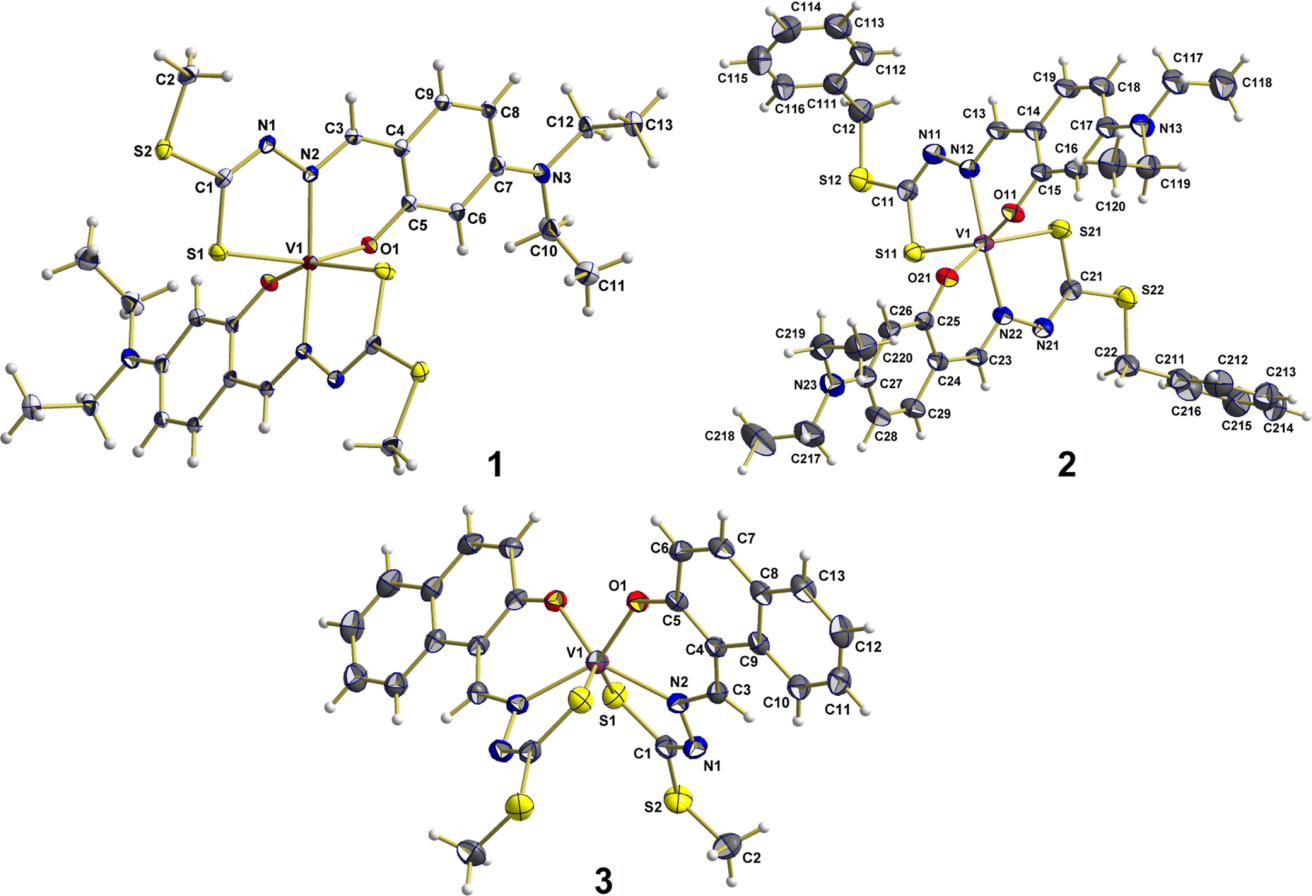
Ball-and-stick models of the discrete, monomeric complexes found in the SC-XRD structures of **1**, **2**, and **3**, showing the atom labeling scheme of the asymmetric unit. All non-hydrogen atoms are drawn as thermal displacement ellipsoids of the 50% level, while hydrogen atoms have been drawn as spheres of arbitrary radii.

The solid-state structures of **1** (monoclinic, *C*2/*c*), **2** (triclinic, *P*1̅), and **3** (monoclinic, *P*2/*n*) were characterized by SC-XRD techniques. An overview of the solid-state conformation of all three complexes is shown in Figure 1, and selected bond distances and angles are given in Table 1. The three complexes belong to three different point groups (**1** = *C*_*i*_, **2** = *C*_1_, and **3** = *C*_2_), thus exhibiting different symmetry elements (**1** = inversion center at the position of the central vanadium atom, **2** = asymmetric, and **3** = a twofold rotation axes through the vanadium atom and in between both ligands).

**Table 1:** Selected Bond Distances and Angles for Complexes **1**, **2,** and **3**

complex	1	2		3
prefix *m* of ligand	not applied	*m* = 1	*m* = 2	not applied
Bond Distances (Å)				
V(1)–O(*m*1)	1.923(2)	1.926(1)	1.937(1)	1.894(2)
V(*m*1)–N(*m*2)	2.047(2)	2.071(2)	2.064(2)	2.104(2)
V(*m*1)–S(*m*1)	2.3524(6)	2.3434(7)	2.3500(7)	2.3519(8)
Bond Angles (°)				
O(*m*1)–V(1)–O(*m*1)^a^	78.27(9)°	78.36(6)°	[−O(2)]	83.3(1)°
S(*m*1)–V(1)–S(*m*1)^a^	108.37(4)°	112.68(3)°	[−S(12)]	138.77(5)°
N(*m*2)–V(1)–N(*m*2)^a^	155.1(1)°	148.04(7)°	[−N(22)]	125.2(1)°
O(*m*1)–V(1)–S(*m*1)	155.6(1)°	148.9(1)°	149.5(1)°	125.3(1)°

a Symmetry operators used to generate equivalent atoms: −*x*+1, *y*, −*z*+1/2 for **1**, −*x*+1/2, *y*, −*z*+1/2 for **3**. For **2,** bond angles are given between chemical equivalents atoms O(1)–O(2), S(11)–S(12), and N(12)–N(22).

The molecular structures of all three compounds contain discrete monomeric vanadium(IV) species coordinated by two tridentate, twofold negative ONS-donor Schiff base ligands. The Schiff bases coordinate to vanadium *via* the O-atom of their deprotonated hydroxyl-OH, the imine N-atom, and the S-atom of the sulfide moieties. In each case, the coordination spheres around the V atoms are strongly distorted. The geometries may be best described as distorted octahedral for **1** and **2** with the O- and S-atoms in *cis-* and the N-atoms in *trans*-positions, while **3** exhibits a distorted trigonal prismatic geometry with the O-atoms in *cis*-positions at one edge of the prism, while the S- and N-atoms are *cis* to each other on the other edges of the prism, as shown in Figure S1.

As a result of these different coordination geometries, bond lengths and angles in the coordination spheres of the vanadium atoms differ significantly (Table 1). Namely, considerable differences are observed in the case of bond angles as a result of different coordination geometries, mostly related to the bond angles between the central vanadium atom and two terminal donors (O, S) atoms of the ligands. In this context, the corresponding values [155.6(1)° for **1** and 148.9(1)°/149.5(1)° for **2**] are typical for the octahedral vanadium coordination with all three donor atoms in a *meridional* arrangement [ideal value: 180°], while the value of 125.3(1)° for **3** corresponds quite well with a trigonal-prismatic vanadium coordination [ideal value of a regular trigonal prism: 125°]. Additionally, bond lengths are also affected. While differences are small (Δ = 0.009 Å) for the vanadium–sulfur distances [d(V–S) range from 2.3434(7) Å for **2** to 2.3524(6) Å for **1**], those for the vanadium–oxygen and vanadium–nitrogen distances are much greater [d(V–O) range from 1.894(2) Å for **3** to 1.937(1) Å for **2** with Δ = 0.043 Å, while d(V–N) range from 2.047(2) for **1** to 2.104(2) for **3** with Δ = 0.057 Å]. To a certain extent, this may be ascribed to the different temperatures at which the crystal structures were determined, but this is not fully consistent with the behavior of **1** (*T* = 100 K) and **2** and **3** (*T* = 296 K). Therefore, other effects must be taken into account. Notably, EPR analysis and DFT simulations indicate that at least two isomers with comparable stability, close to the *meridional* and *facial* limit, coexist in the solutions, from where the solid complexes are isolated (*vide infra*); thus, the isomeric selection experimentally observed could be ascribed to stabilizing crystal packing effects. Here, it is important to highlight that the characterized isomers are defined referring to the limit cases represented by *meridional* and *facial* arrangement, even though complexes **1**–**3** correspond to intermediate structures.

In this context, we highlight two features of the crystal structures of **1**–**3**. The first one concerns the different conformations of the two crystallographic independent ligands found in the crystal structure of **2**, indicated with *m* in Table 1, as they show high conformation flexibility within the ligands (Figure S2), not only in the domain of the sulfide moiety with their low rotation barriers around the sulfur–carbon single bonds, but to a lesser extent also associated to the conjugated double bond system, which obviously may adopt different orientations for coordination.

A second feature is found in the crystal structure of **1** where the formation of dimeric centrosymmetric supramolecules results from the π-interaction of their phenyl moieties (Figure S3), which are almost coplanar to each other with six intermolecular carbon–carbon distances ranging from 3.358 to 3.411 Å. Moreover, similar intermolecular distances (3.257 Å) are found for the nitrogen and carbon atoms attached to the phenyl groups.

### UV–vis Spectroscopy

**Figure 2:**
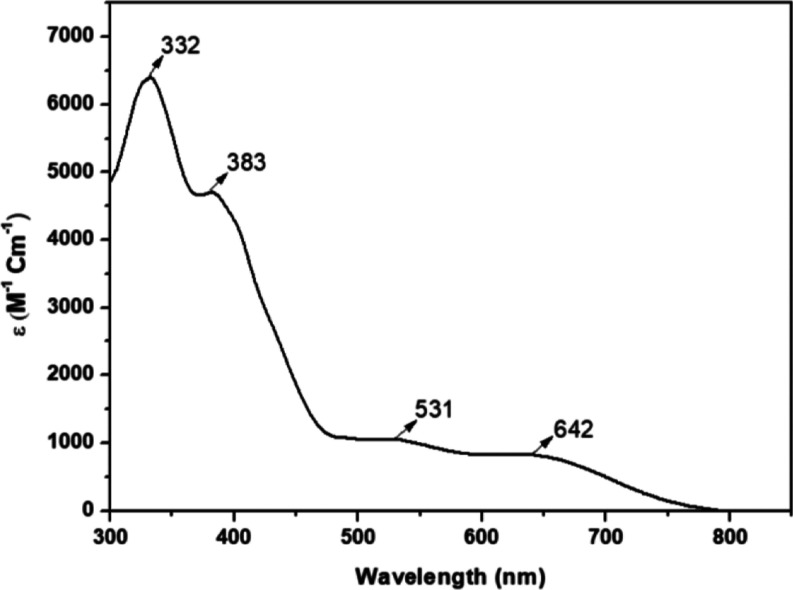
UV–vis spectrum of [V^IV^(L^3^)_2_] (**3**) in DMSO recorded with a concentration of 1.5 × 10^–4^ M and path length = 1 cm.

Electronic absorption spectra of **1**–**4** were recorded in DMSO and show similarities. As a representative example, the UV–vis spectrum of **3** is shown in Figure 2, and those of **1**, **2,** and **4** are reported in Figure S4. In the wavelength range 330–650 nm, three absorptions were observed for complexes **1** and **2**, whereas four bands are detected for **3** and **4**. As pointed out in the literature, for non-oxido vanadium(IV) complexes, the UV–vis spectrum is dominated by ligand-to-metal charge transfer (LMCT) transitions, with large values of the molar absorption coefficient (generally, ε > 1000 M^–1^ cm^–1^ in the visible and ε > 5000 M^–1^ cm^–1^ in the UV region), from MOs centered on the ligands to V-d based orbitals.^53,128^ TD-DFT calculations carried out on **1**–**4** confirmed these findings and indicated that all the absorption bands are strongly mixed and correspond to a combination of various electronic transitions.

In the visible region, the absorptions in the range 624–642 nm (ε = 159–844 M^–1^ cm^–1^) and 530–532 nm (ε = 265–1080 M^–1^ cm^–1^) correspond to excitations from ligand-centered MOs, as from highly delocalized π orbitals (for example, L^1^-π and L^1’^-π) to V-d_*z*_^2^ or V-d_*xz*_.^65^ The bands at 398 nm for **1** and **2** (with molar absorption coefficients of 7966 and 6557 M^–1^ cm^–1^, respectively) and between 332 and 384 nm for **3** and **4** (with molar absorption coefficients of range of 6402–4707 M^–1^ cm^–1^) are countersigned by values of ε significantly higher than 4000 M^–1^ cm^–1^.^65^ They are LMCT absorptions mixed with ligand-to-ligand charge transfers. The principal transitions involve MOs with characters V-d_*z*_^2^, V-d_*xz*_, and V-d_*yz*_ for vanadium and MOs with π character for the ligands.

### ESI-Mass Spectroscopy

**Figure 3:**
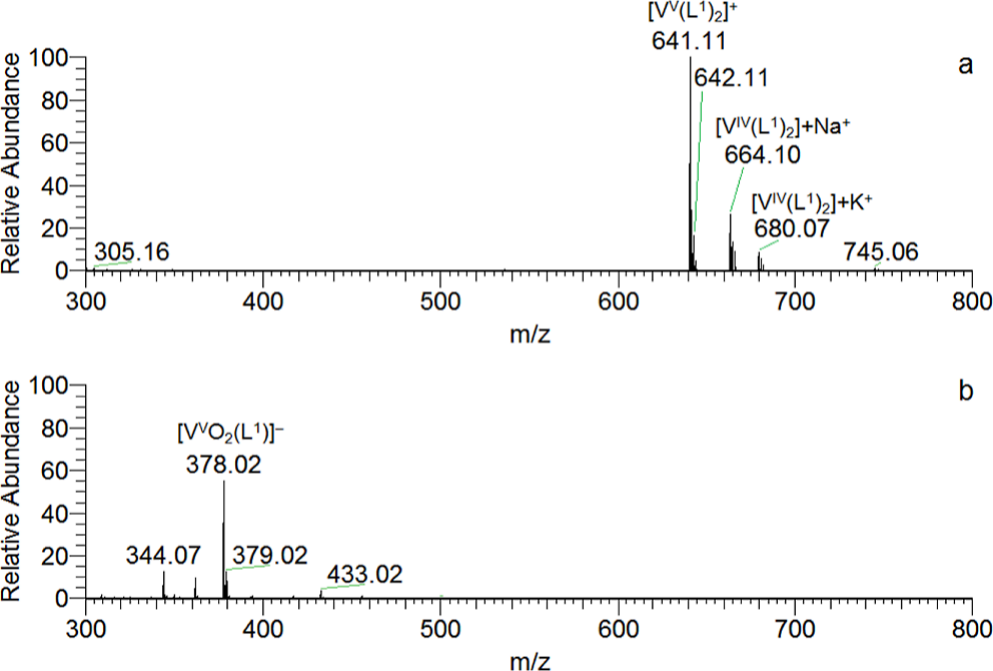
ESI-MS spectra in the positive-ion mode (a) and negative-ion mode (b) recorded on complex **1** dissolved in CH_3_CN (50 μM).

ESI-MS spectra of the compounds **1**–**4** were recorded in several solvents or mixtures of solvents: CH_3_CN, DMSO, MeOH/H_2_O 90/10 (v/v), and DMSO/H_2_O 90/10 (v/v) in the positive- and negative-ion mode. The ESI-MS(+) and ESI-MS(−) spectra of **1** in CH_3_CN, recorded with a vanadium concentration of 50 μM, are reported in Figure 3. In the positive-ion mode spectrum, the major peaks are at *m*/*z* = 641.11, 642.11, 664.10, and 680.07. No peaks ascribable to the free ligand were detected, and this indicates the stability of the complex to hydrolysis even at these low metal concentrations. The peak at *m*/*z* 641.11 is assigned to the oxidized species [V^V^(L^1^)_2_]^+^ (simulation in panel a of Figure S5), while that at *m*/*z* 642.11 can be explained with [V^IV^(L^1^)_2_] + H^+^ and/or to the isotopic pattern of [V^V^(L^1^)_2_]^+^ (panels b and c of Figure S5). The high-resolution instrument used in this work allowed us to distinguish between the two options; even though the simulation seems to be identical up to two decimal figures, it is significantly different when four decimal figures are considered. In particular, a peak at *m*/*z* 642.1094 is expected for [V^V^(L^1^)_2_]^+^ and at *m*/*z* 642.1138 for [V^IV^(L^1^)_2_] + H^+^, which therefore contribute to the signals in the range *m*/*z* 641–645 in a negligible mode (Figure S6). To the best of our knowledge, this is one of the first cases in which ESI-MS allows to discriminate unambiguously two complexes in two different metal oxidation states. Instead, the signals at *m*/*z* 664.11 and at *m*/*z* 680.07 can be assigned without any ambiguity to the adducts [V^IV^(L^1^)_2_] + Na^+^ and [V^IV^(L^1^)_2_] + K^+^ (simulations in Figures S7 and S8). The values of *m*/*z* for all the species detected by ESI-MS are listed in Table 2. Summarizing the results, [V^IV^(L^1^)_2_] (**1**) can undergo partial oxidation to [V^V^(L^1^)_2_]^+^.

**Table 2:** Identified Species in the Positive- and Negative-Ion Mode ESI-MS Spectra of Complexes **1**–**4**^a^

ion	composition	experimental *m*/*z*^b^	calculated *m*/*z*^b^	error (ppm)^c^
[V^V^(L^1^)_2_]^+^	C_26_H_34_N_6_O_2_S_4_V	641.1066	641.1060	0.9
[V^IV^(L^1^)_2_] + Na^+^	C_26_H_34_N_6_NaO_2_S_4_V	664.0960	664.0958	0.3
[V^IV^(L^1^)_2_] + K^+^	C_26_H_34_KN_6_O_2_S_4_V	680.0699	680.0697	0.3
[V^V^O_2_(L^1^)]^−^	C_13_H_17_N_3_O_3_S_2_V	378.0161	378.0156	1.3
[V^V^(L^2^)_2_]^+^	C_38_H_42_N_6_O_2_S_4_V	793.1688	793.1686	0.3
[V^IV^(L^2^)_2_] + Na^+^	C_38_H_42_N_6_NaO_2_S_4_V	816.1585	816.1584	0.1
[V^IV^(L^2^)_2_] + K^+^	C_38_H_42_KN_6_O_2_S_4_V	832.1323	832.1323	0.0
[V^V^O_2_(L^2^)]^−^	C_19_H_21_N_3_O_3_S_2_V	454.0480	454.0469	2.4
[V^V^(L^3^)_2_]^+^	C_26_H_20_N_4_O_2_S_4_V	598.9911	598.9903	1.3
[V^IV^(L^3^)_2_] + Na^+^	C_26_H_20_N_4_NaO_2_S_4_V	621.9814	621.9801	2.1
[V^IV^(L^3^)_2_] + K^+^	C_26_H_20_KN_4_O_2_S_4_V	637.9561	637.9540	3.3
[V^V^O_2_(L^3^)]^−^	C_13_H_10_N_2_O_3_S_2_V	356.9586	356.9578	2.2
[V^V^(L^4^)_2_]^+^	C_38_H_28_N_4_O_2_S_4_V	751.0533	751.0529	0.5
[V^IV^(L^4^)_2_] + Na^+^	C_38_H_28_N_4_NaO_2_S_4_V	774.0427	774.0413	1.8
[V^V^O_2_(L^4^)]^−^	C_19_H_14_N_2_O_3_S_2_V	432.9899	432.9891	1.8

a The data are referred to the mass spectra recorded in CH_3_CN.

b Experimental and calculated *m*/*z* values refer to the monoisotopic peak with the highest intensity.

c Error in ppm with respect to the experimental value, calculated as 10^6^ × [Experimental (*m*/*z*) – calculated (*m*/*z*)]/calculated (*m*/*z*).

The negative-ion spectrum shows few signals and a major peak at *m*/*z* = 378.02 (Figure 3b). The simulations allowed us to determine the stoichiometry of this species which is assigned to [V^V^O_2_(L^1^)]^−^, formed upon the oxidation of [V^IV^(L^1^)_2_]. The simulation of the isotopic pattern is represented in Figure S9.

The behavior of complexes **2**–**4** is comparable, and the observed species are listed in Table 2. The results in the mixture MeOH/H_2_O 90/10 (v/v) are globally similar, but it should be mentioned that the presence of water decreases the solubility of the complexes and favors the oxidation to V^V^, as shown in Figure S10.

A few aspects deserve to be emphasized: (i) the possibility that the observed oxidation process partially occurs in-source, during the recording of the spectra, cannot be excluded;^120,129^ (ii) the tridentate coordination of the ONS ligand to V is quite strong, and the hydrolysis occurs at low extension, both in CH_3_CN and in MeOH/H_2_O 90/10 (v/v), even at the low metal concentrations used for recording the spectra; (iii) a non-oxido vanadium(V) species is formed in solution; (iv) the formation of non-oxido vanadium(V), a hard metal ion, is rather unusual and few examples have been reported in the literature up to now;^130,131^ and (v) under our experimental conditions, [V^IV^(L^1^)_2_] forms adducts only with Na^+^ and K^+^ ions but not with H^+^.

### NMR Spectroscopy

Proton NMR spectra of H_2_L^1–4^ were recorded in DMSO-*d*_6_. These spectra exhibit a resonance in the range δ = 13.46–13.13 ppm attributable to −NH groups,^132^ one singlet peak of the phenolic −OH groups in the region δ = 11.09–10.08 ppm due to intermolecular hydrogen bonding, and another sharp singlet at δ = 9.20–8.31 ppm due to the azomethine −CH protons.^53,64,89,127,132−138^ The aromatic proton signals of the free ligands are clearly observed in the expected range between δ 8.80 and 6.07 ppm. For the aliphatic protons, two multiplets are observed in the range δ = 3.36–3.31 ppm for −N–CH_2_ and δ = 1.08–1.11 ppm for the −CH_3_ protons of the diethylamino fragment in the case of H_2_L^1–2^. Singlets for the CH_2_S– protons of H_2_L^2^ and H_2_L^4^ are observed around ∼4.45 ppm, which are absent in the spectra of H_2_L^1^ and H_2_L^3^.^132^ Compounds H_2_L^1^ and H_2_L^3^ contain one extra singlet in the aliphatic region near 2.51 ppm due to the −SCH_3_ protons.^53,126^

### Electrochemical Studies

**Figure 4:**
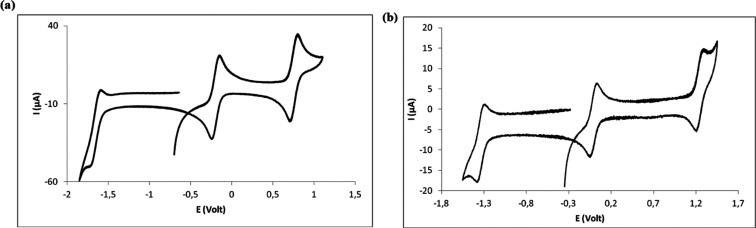
Cyclic voltammograms of (a) [V^IV^(L^2^)_2_] (**2**) and (b) [V^IV^(L^4^)_2_] (**4**) obtained from Bu_4_NBF_4_/CH_2_Cl_2_ (0.10 M).

The cyclic voltammograms of **1**–**4** were obtained from Bu_4_NBF_4_/CH_2_Cl_2_ (0.10 M) electrolyte solutions under dinitrogen at a scan rate of 200 mV s^–1^. The potentials were measured in Volt (±10 mV) *vs* SCE using [Fe(η^5^-C_5_H_5_)_2_]^0/+^ (*E*_1/2_^*ox*^ = 0.475 V) as an internal reference. The redox potential data are summarized and displayed in Table 3. All complexes present reversible anodic waves in the range 0.74–1.18 V, attributable to the V^IV^ → V^V^ oxidation processes and cathodic reversible waves from −0.08 to −0.20 V, assignable to the V^IV^ → V^III^ reduction^64^ (Table 3). One additional *quasi*-reversible lower potential cathodic wave is observed from −1.39 to −1.73 V, which is attributed to a ligand-centered reduction process. Such an attribution is based on the study of the redox properties of H_2_L^3^ and H_2_L^4^ as representative examples (Table 3 and Figure S11). The cyclic voltammograms obtained for the free ligands display irreversible cathodic processes with potentials that differ from those of the related complexes by *ca*. 5 mV or less (Table 3). In the complexes, such cathodic waves gain reversibility, a behavior earlier reported for the similar types of non-oxido vanadium(IV) complexes.^53,65^ Such a behavior suggests that the metal ion partially accommodates the increase in electron density without further changes in the ligand. The reversibility of the anodic and cathodic processes shows that no major structural modifications occur upon electron transfer from or to the hexa-coordinated V^IV^ complexes as observed for several other non-oxido V^IV^ species.^139,140^ Representative cyclic voltammograms of complexes **2** and **4** are displayed in Figure 4.

**Table 3:** Cyclic Voltammetry Data for **1**–**4** and H_2_L^3–4^^a^^,^^b^

complex/ligand	^II^*E*_1/2_^red^	^I^*E*_1/2_^red^	*E*_1/2_^*ox*^
1	–1.68	–0.20	0.75
2	–1.73	–0.20	0.74
3	–1.49	–0.12	1.10
4	–1.39	–0.08	1.18
H_2_L^3^	–1.47^c^		1.27
H_2_L^4^	–1.34^c^		1.33

a Values in Volt (±10 mV) *vs* SCE measured at a scan rate of 200 mV/s.

b In Bu_4_NBF_4_/CH_2_Cl_2_ (0.10 M).

c *E*_*p*_^red^.

The electrochemical behavior of **1**–**4** is similar to those of other non-oxido V^IV^ complexes containing of ONS and ONO donor ligand systems reported earlier,^53,65^ where rather similar reversible oxidation and reduction processes (V^IV^ → V^V^ and V^IV^ → V^III^) were observed. Additionally, **1** and **2,** which have diethylamine substituents at the phenyl ring (X = NEt_2_; Scheme 1), display considerably lower V^IV^ → V^V^ oxidation potentials (Table 3) than the unsubstituted V^IV^ complexes (**3** and **4**). Such a trend is attributed to the high electron releasing character of the amine group in agreement with the pattern previously reported for V^IV^ species formed by the phenyl amine (X = NH_2_)-substituted ONO ligands that also display lower potentials than their analogues.^65^ For **3** and **4,** the anodic processes occur at potentials in the same range (1.1 V *vs* SCE) of the formerly reported dithiocarbazate species.^53^ For **1–4,** the higher potential cathodic process occurs within a short range of potentials (from −0.2 to 0.0 V) either for dithiocarbazate or for aroylhydrazone complexes, in agreement with the V^IV^ → V^III^ reduction being less sensitive to the characteristics of the substituents at the ligand than the V^IV^ → V^V^ oxidation.

### Optimization of the Stable Structures in Solution

On the basis of the results obtained with single-crystal X-ray diffraction and ESI-MS data, the species stable in media containing moderate amounts of water are [V^IV^(L^1–4^)_2_], *i.e.* the same species isolated in the solid state, and [V^V^(L^1–4^)_2_]^+^ and [V^V^O_2_(L^1–4^)]^−^, derived from the partial oxidation of **1**–**4**.

**Figure 5:**
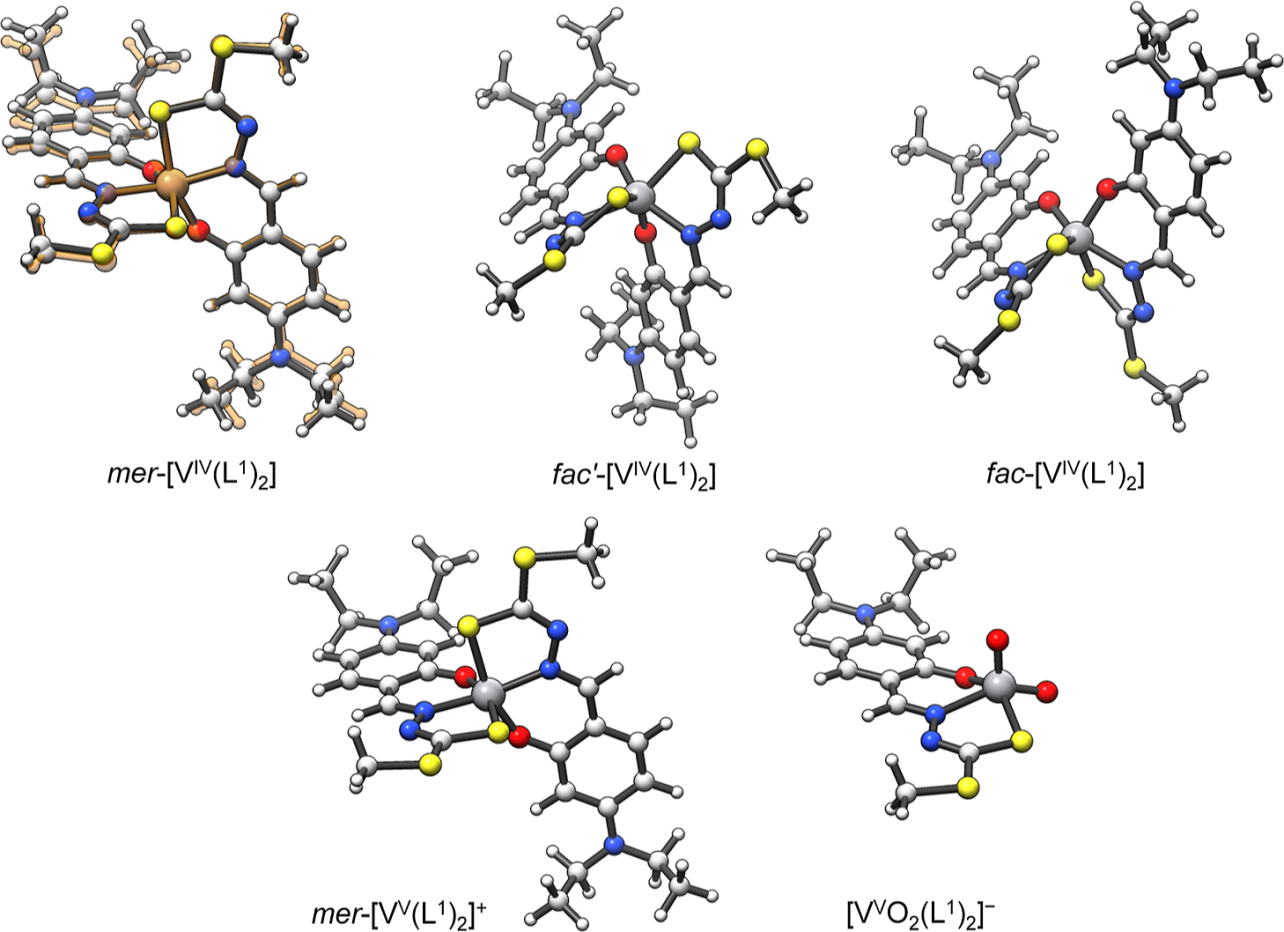
DFT-optimized structures of *mer*-[V^IV^(L^1^)_2_], *fac’*-[V^IV^(L^1^)_2_], *fac*-[V^IV^(L^1^)_2_], *mer*-[V^V^(L^1^)_2_]^+^, and [V^V^O_2_(L^1^)]^−^. For *mer*-[V^IV^(L^1^)_2_] isomer (**1**), the superposition with the SC-XRD structure (in orange) is also shown.

The structures of the complexes formed in solution were optimized by DFT methods following the procedure reported in the literature.^111,112^ As an example, those of [V^IV^(L^1^)_2_], [V^V^(L^1^)_2_]^+^, and [V^V^O_2_(L^1^)]^−^ are depicted in Figure 5. Selected bond lengths and angles of all the optimized structures are collected in Tables S2–S4. Notably, the prediction of the geometric parameters is very good, as demonstrated by the superposition of the experimental and calculated structures of complex **1** (Figure 5, top left).

It is interesting to observe that for [V^IV^(L^1–4^)_2_] (**1**–**4**), three different energy minima were found, one corresponding to the *meridional* arrangement of the ligands and two to the *facial* one. In Table 4, the angles between the external donors of the same ligand molecule are indicated; they should be 180° and 90° for *mer* and *fac* situations, respectively. Notably, for **1** and **2** complexes, the *mer* isomer is the most stable, while for **3** and **4,** it is the *fac* isomer. This agrees well with the SC-XRD structures that show the same trend: in fact, the geometry of **1** and **2** is close to the *meridional* limit and that of **3** to the *facial* limit (see section “Single-Crystal X-ray Diffraction Analysis of Complexes **1–3**”). The flexible structure of the ligands accounts for the coexistence of the two isomers. The free energy difference between the three isomers is very low, between 0.1 and 2.3 kcal mol^–1^ (Table 4); so, it is possible that more than one species exists in solution and subtle factors such as the solubility, the interaction between the solid complexes, and the crystal packing could favor the formation of one of the three isomers in the solid state.

**Table 4:** Bond Angles and Relative Δ*G* of Formation in Solution (ΔΔ*G*_aq_) for the Isomers of Non-Oxido Complexes [V^IV^(L^1–4^)_2_]

	*trans* angles^b^^,^^c^			
isomer^a^	N_1_–V–N_2_	O_1_–V–S_1_	O_2_–V–S_2_	ΔΔ*G*_aq_^d^
***mer*-[V**^**IV**^**(L**^**1**^**)**_**2**_**]**^a^	153.9 (155.1)	156.3 (155.6)	156.3 (155.6)	0.0
*fac*-[V^IV^(L^1^)_2_]	121.4	123.1	120.6	0.7
*fac’*-[V^IV^(L^1^)_2_]	118.8	124.6	124.6	1.7
***mer*-[V**^**IV**^**(L**^**2**^**)**_**2**_**]**^a^	154.0 (148.0)	156.4 (148.9)	156.6 (149.5)	0.0
*fac*-[V^IV^(L^2^)_2_]	122.0	123.4	120.9	0.1
*fac’*-[V^IV^(L^2^)_2_]	118.8	124.6	124.6	1.0
*mer*-[V^IV^(L^3^)_2_]	161.7	164.0	164.0	0.9
***fac*-[V**^**IV**^**(L**^**3**^**)**_**2**_**]**^a^	121.1 (125.2)	122.0 (125.2)	119.7 (125.2)	0.0
*fac’*-[V^IV^(L^3^)_2_]	122.9	124.8	124.8	1.8
*mer*-[V^IV^(L^4^)_2_]	161.2	163.4	163.4	1.1
***fac*-[V**^**IV**^**(L**^**4**^**)**_**2**_**]**^a^	121.2	122.0	119.8	0.0
*fac’*-[V^IV^(L^4^)_2_]	161.7	124.7	124.7	2.3

a In bold, the most stable isomers in aqueous solution.

b Values in degrees.

c In parentheses, the experimental values extracted from SC-XRD structures.

d Values in kcal mol^–1^.

Concerning [V^V^(L^1–4^)_2_]^+^, the structures are very similar to [V^IV^(L^1–4^)_2_], in agreement with the electrochemical results that indicate a reversible anodic wave for **1**–**4**, while [V^V^O_2_(L^1–4^)]^−^ are typical dioxidovanadium(V) complexes with a geometry intermediate between the square pyramid and the trigonal bipyramid.

### EPR Spectroscopy

**Figure 6:**
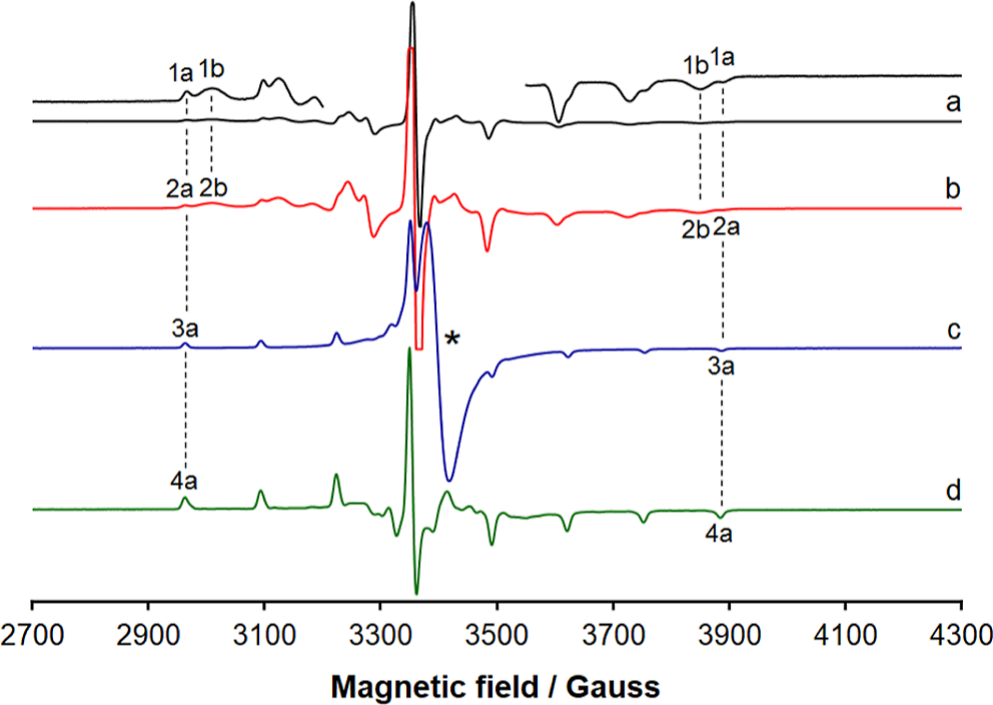
First derivative X-band anisotropic EPR spectra of (a) [V^IV^(L^1^)_2_] (**1**); (b) [V^IV^(L^2^)_2_] (**2**); (c) [V^IV^(L^3^)_2_] (**3**); and (d) [V^IV^(L^4^)_2_] (**4**). All the spectra were recorded in CH_2_Cl_2_ at 120 K with an approximate vanadium concentration of 8 × 10^–4^ M. With the dotted lines, the *M*_I_ = −7/2, 7/2 resonances of the species **1a–4a** and **1b–2b** are indicated, while with the asterisk, the isotropic absorption due to the undissolved solid complex **3** is shown. The low- and high-field regions of the spectrum of **1** are amplified by 20 and 25 times, respectively, at the top of the figure.

EPR spectra of complexes **1**–**4** were recorded at 120 K after dissolution in dichloromethane. Spin Hamiltonian parameters (factor *g* and ^51^V hyperfine coupling constant *A*) were extracted simulating the spectra with WinEPR software^141^ and were compared with the experimental ones. They are reported in Table 5, while the experimental spectra of **1**–**4** are represented in Figure 6.

**Table 5:** Experimental (Exptl) and DFT Calculated (Calcd) Spin Hamiltonian EPR Parameters for the Non-Oxido V^IV^ Complexes [V^IV^(L^1–4^)_2_]

isomer	*g*_x_^exptl^	*g*_y_^exptl^	*g*_z_^exptl^	*A*_x_^exptl^^a^	*A*_y_^exptl^^a^	*A*_z_^exptl^^a^	*A*_z_^calcd^^a^^,^^b^	PD (*A*_z_)^c^
*mer*-[V^IV^(L^1^)_2_]	1.987	1.980	1.960	–10.0	–36.0	–110.2	–104.6	–5.1
*fac*-[V^IV^(L^1^)_2_]	1.987	1.982	1.961	–12.0	–38.4	–120.6	–120.2	–0.3
*fac’*-[V^IV^(L^1^)_2_]							–115.6	–4.2
*mer*-[V^IV^(L^2^)_2_]	1.986	1.980	1.960	–9.8	–35.7	–110.0	–104.4	–5.1
*fac*-[V^IV^(L^2^)_2_]	1.986	1.982	1.961	–11.8	–38.1	–120.7	–120.2	–0.4
*fac’*-[V^IV^(L^2^)_2_]							–115.6	–4.2
*mer*-[V^IV^(L^3^)_2_]	d	d	d	d	d	d	–98.2	d
*fac*-[V^IV^(L^3^)_2_]	1.986	1.981	1.962	–11.7	–38.4	–120.9	–120.0	–0.7
*fac’*-[V^IV^(L^3^)_2_]							–116.7	–3.5
*mer*-[V^IV^(L^4^)_2_]	d	d	d	d	d	d	–97.2	d
*fac*-[V^IV^(L^4^)_2_]	1.986	1.982	1.962	–12.0	–38.2	–120.6	–120.0	–0.5
*fac’*-[V^IV^(L^4^)_2_]							–116.7	–3.2

a Values in 10^–4^·cm^–1^.

b Calculated at the B2PLYP/6-311g(d,p) DFT theory level.

c Percent deviation (PD) calculated as 100 × [(|*A*_z_|^calcd^ – |*A*_z_|^exptl^)/|*A*_z_|^exptl^].

d Isomer not present in solution.

It is noteworthy that, when **3** and **4** are dissolved in CH_2_Cl_2_, only one set of resonances is detected (species **3a** and **4a** in the traces c and d) with a value of *A* between 120 and 121 × 10^–4^ cm^–1^. In contrast, for **1** and **2**, two complexes are observed, the first denoted by **1a** and **2a,** which coincide with those revealed in the systems containing **3** and **4**, and the second ones denoted by **1b** and **2b**, with a smaller *A* constant, around 110 × 10^–4^ cm^–1^. The values of *A*, much lower than (140–145) × 10^–4^ cm^–1^, are typical of non-oxido vanadium(IV) complexes.^114^ Considering the results from X-ray diffraction analysis that indicate that two types of crystal structures are obtained, one close to the limit of the *meridional* isomerism (**1** and **2**) and another close to the *facial* arrangement (**3**, see section “Single-Crystal X-ray Diffraction Analysis of Complexes **1–3**”), we propose that the resonances belong to the two isomeric forms of **1**–**4**, those corresponding to the *fac* and *mer* structures.

**Figure 7:**
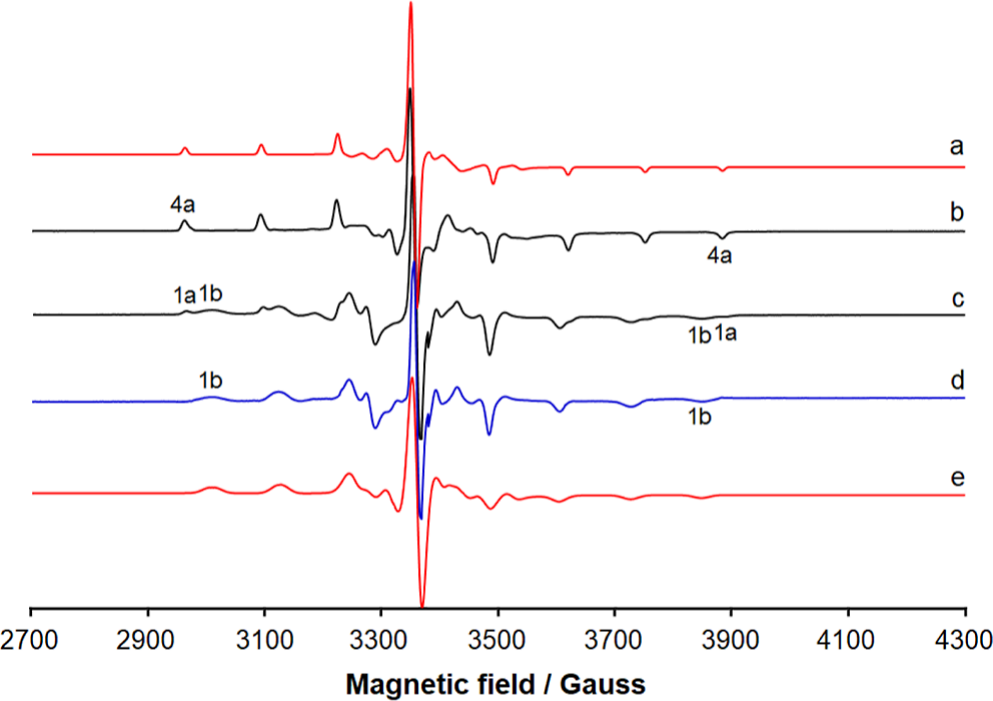
First derivative X-band anisotropic EPR spectra of (a) simulated spectrum of **4a** obtained with WinEPR software; (b) experimental spectrum of [V^IV^(L^4^)_2_] (**4**); (c) experimental spectrum of [V^IV^(L^1^)_2_] (**1**); (d) spectrum of the species **1b** obtained from the experimental spectrum of **1** subtracting the signals of **1a**; (e) simulated spectrum of **1b**. With **1a** and **4a,** the *M*_I_ = −7/2, 7/2 resonances of the *fac* isomers are indicated, while **1b** denotes the *M*_I_ = −7/2, 7/2 resonances of *mer* isomer. The experimental spectra were recorded in CH_2_Cl_2_ at 120 K with an approximate vanadium concentration of 8 × 10^–4^ M.

In Figure 7, the experimental spectra of **1** and **4** (traces b and c) are compared with the simulated ones. Notably, the spectrum of the pure species **1b** can be obtained subtracting the signals of **1a** from the experimental spectrum of **1** and can be satisfactory simulated with WinEPR software (Table 5).

The ***A*** tensor of the ^51^V nucleus for non-oxido V^IV^ complexes was computed for each structure stable in solution (isomers *mer*, *fac*, and *fac’*; see the previous section) through the method implemented into the ORCA package,^113,142,143^ using the functional B2PLYP coupled with the triple-ζ basis 6-311g(d,p) (Table 5). The B2PLYP/6-311g(d,p) combination gives high quality predictions of the largest component of the ***A*** tensor.^114^ The theory background was described elsewhere.^144^*A*_x_, *A*_y_, and *A*_z_ are negative, as expected for V^IV^ species, but in the text, the absolute values are reported for convenience of discussion.

By examining the data in Table 5, it emerges that the order of the constant |*A*_z_| is *fac* ∼ *fac’* > *mer*, in agreement with what was experimentally observed. The prediction, expressed as percent deviation, is in the range 0–5%, in line with the data previously published; in particular, in all the cases, a slight underestimation of |*A*_z_| is expected.^114^ It must be observed that for **3** and **4,** only the *fac* isomer should exist in solution having the large value of the hyperfine coupling constant (species **3a** and **4a** in Figure 6). This is in line with the X-ray diffraction determinations and DFT calculations, which suggest that, for these two complexes, the *facial* arrangement is more stable than the *meridional* one (Table 4). Instead, with **1** and **2**, the two sets of resonances could be assigned to *fac* (species **1a** and **2a**) and *mer* isomers (**1b** and **2b**), in agreement with SC-XRD and DFT data. Notably, the existence of the two isomers in solution, *meridional* and *facial*, has been proposed by Chaudhuri and co-workers for non-oxido V^IV^ species formed by some ONO ligands.^145^

### Solution Stability of Complexes **1**–**4**

The stability of all complexes was established in water containing solvents through UV–vis, ESI mass spectrometry, and NMR and EPR spectroscopy.

#### UV–vis Studies

Solutions of complexes **1**–**4** were prepared in a mixture DMSO/H_2_O 10/90 (v/v) with a vanadium concentration of 1 mM. Their UV–vis spectra are depicted in Figure S12. The shape of the spectrum of **1** in this solvent mixture is very similar to the one measured in DMSO; therefore, we can conclude that the same type of coordination is retained.

When complex **1** was dissolved in a solution containing minimum essential medium (MEM, which does not contain FBS) instead of water, precipitation was evident from the beginning (Figure S13).

#### ESI-MS Studies

The aged solutions in MeOH/H_2_O 90/10 (v/v) were diluted in CH_3_CN and analyzed by ESI-MS (concentration 50 μM). For all complexes, the most important species remained those detected at *t* = 0 h, *i.e.,* the non-oxido V^IV^ complexes [V^IV^(L^1–4^)_2_]; the V^V^ species [V^V^(L^1–4^)_2_]^+^, derived from the oxidation of the previous one, and [V^V^O_2_(L^1–4^)]^−^ were also detected. At these experimental conditions, the non-oxido V^IV^ complexes seem to be stable after 24 h with a slight increase in the oxidation process, as indicated by the increment of the relative amount of [V^V^O_2_(L^1^)_2_]^−^ (Figure S14). This demonstrates that, under the biological experiments, the active species may be one or more of those present in solution: [V^IV^(L^1–4^)_2_], [V^V^(L^1–4^)_2_]^+^, and/or [V^V^O_2_(L^1–4^)]^−^. This was considered during the docking studies.

#### EPR Studies

**Figure 8:**
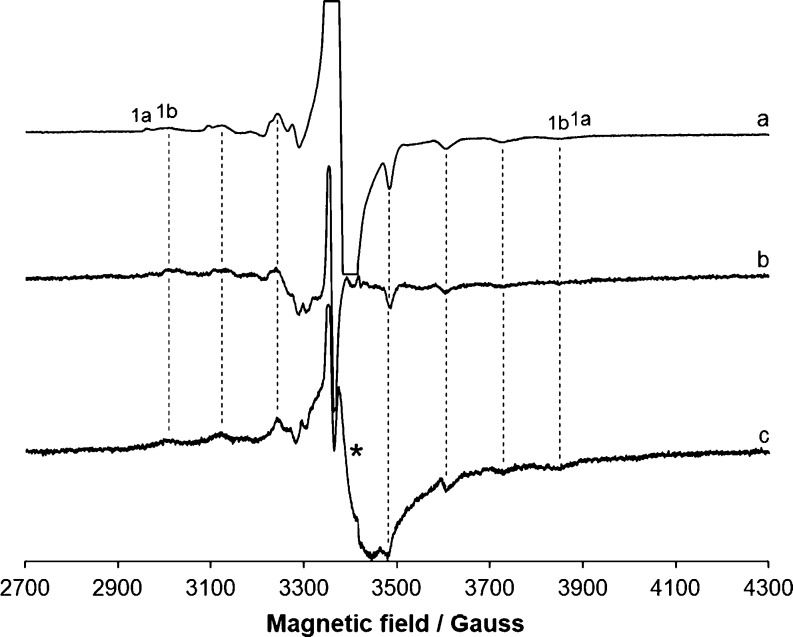
First derivative X-band anisotropic EPR spectra of (a) complex **1** dissolved in DMSO; (b) complex **1** dissolved in DMSO after 24 h; and (c) complex **1** dissolved in the phosphate buffer (pH 7.4) after 24 h. *Fac* and *mer* isomers are denoted with **1a** and **1b**, while with the dotted lines the *M*_I_ = −7/2, −5/2, −3/2, 1/2, 3/2, 5/2, 7/2 resonances of the species **1b** are shown. The presence of a minor amount of the undissolved solid causes the isotropic resonance centered around 3400 Gauss and indicated by the asterisk. The approximate vanadium concentration to record the spectra was 8 × 10^–4^ M.

EPR spectra were recorded 24 h after the dissolution of the most soluble compound, [V^IV^(L^1^)_2_] (**1**), in DMSO and in an aqueous solution in phosphate buffer at pH 7.4 (Figure 8). It must be observed that complex **1** is not completely soluble in phosphate buffer, but its solubility is enough to record a well-resolved EPR signal. The spectrum in DMSO is very similar to that in CH_2_Cl_2_ (cfr. traces in Figures 6 and 8), with the coexistence of *fac* (**1a**) and *mer* (**1b**) isomers. When the spectrum is recorded after 24 h, the pattern remains substantially unaltered, even though a significant decrease in the spectral intensity is detected (trace b of Figure 8). In aqueous solution, the behavior is more or less the same, and after 24 h, the resonances of [V^IV^(L^1^)_2_] are clearly visible (trace c of Figure 8).

The decrease of the signal intensity over time can be related to the oxidation of V^IV^ to V^V^ and the formation of the species [V^V^(L^1^)_2_]^+^ and [V^V^O_2_(L^1^)_2_]^−^, as revealed in the ESI-MS experiments described above.

### BSA Binding Study

#### Fluorescence Competition Titrations of **1**–**4** with BSA

**Figure 9:**
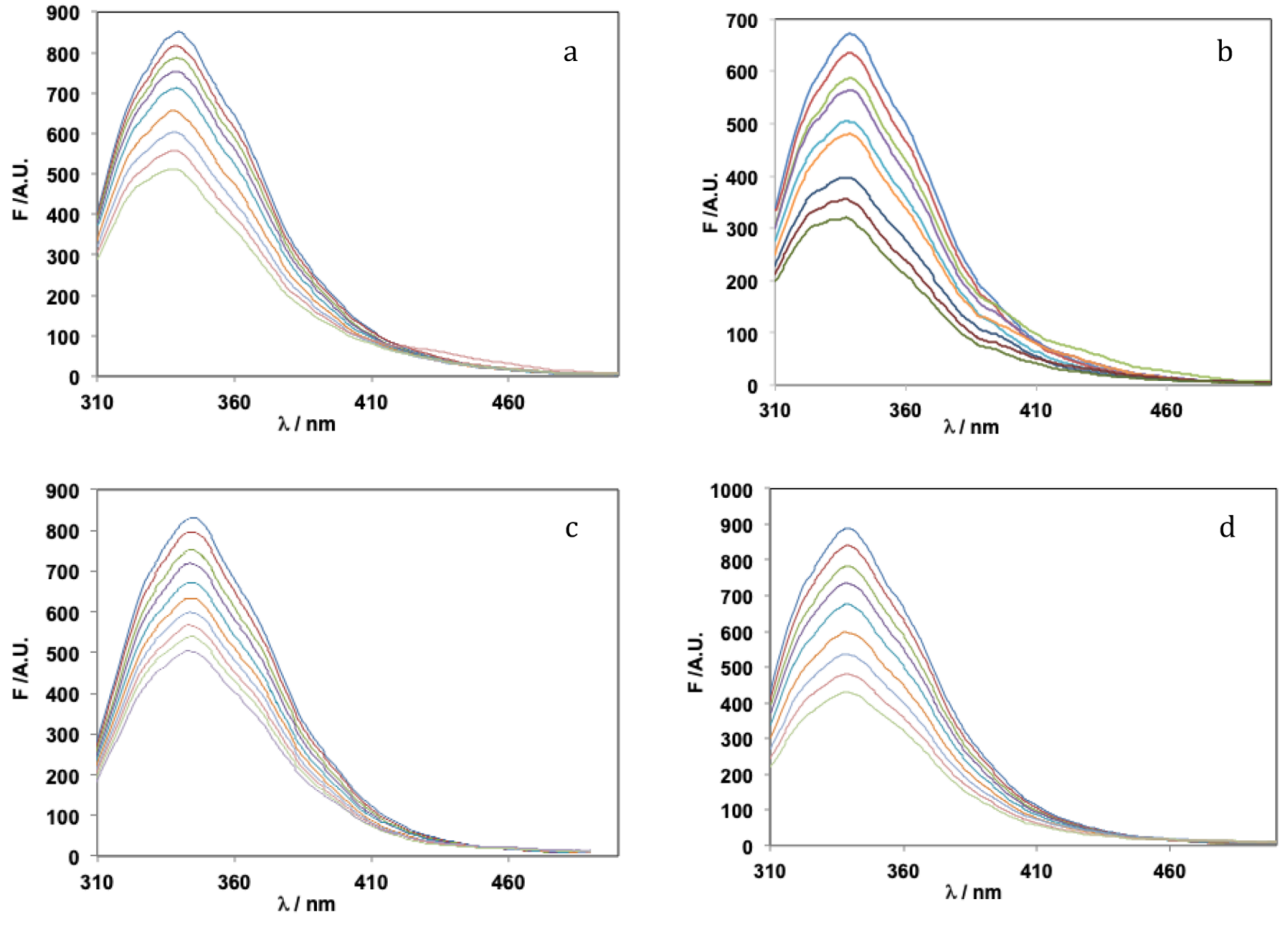
Fluorescence emission titration spectra of BSA, measured at room temperature: (a) **1** with [BSA] = 1.39 μM and [**1**]:[BSA] = 0.0–2.1; (b) **2** with [BSA] = 1.15 μM and [**2**]:[BSA] = 0.0–3.4; (c) **3** with [BSA] = 1.35 μM, and [**3**]:[BSA] = 0.0–3.4; and (d) **4** with [BSA] = 1.33 μM and [**4**]:[BSA] = 0.0–2.9. In all systems, progressive quenching (emission intensity decrease) was observed with the addition of the solutions containing **1**–**4**.

Fluorescence titration data for all systems are depicted in Figure 9. In all cases, significant quenching of BSA fluorescence is observed, due to its Trp residues, but no major spectral shifts were observed. As concluded in the next section on docking studies (*vide infra*), the binding of complexes may take place close to the Trp213 residue, this explaining the strong quenching of fluorescence experimentally observed.

**Figure 10:**
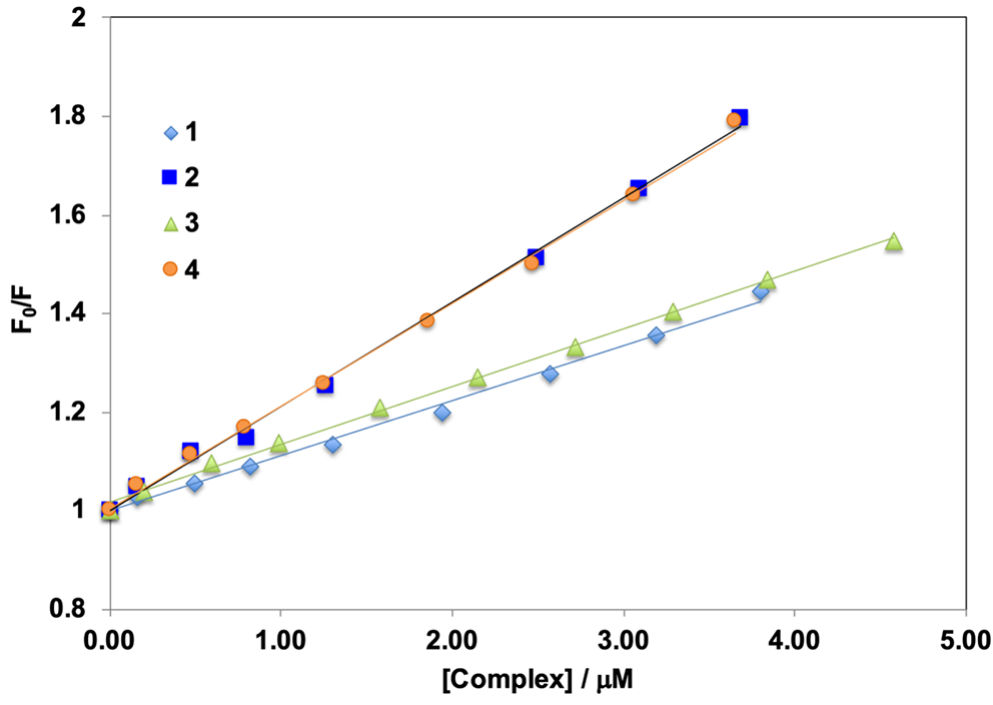
Stern–Volmer plots (*vs* [*Q*]) for complexes **1**–**4**.

Stern–Volmer quenching constants as well as the fitting parameter (*r*^2^) were determined by assuming that the species present in solution are the complexes [V^IV^(L^1–4^)_2_]; the results are included in Table 6. Figure 10 shows the Stern–Volmer plots for all systems. Complexes **1** and **3** show *K*_SV_ constants of (1.1–1.2) × 10^5^ M^–1^, while for **2** and **4,***K*_SV_ are *ca.* 2.1 × 10^5^ M^–1^, confirming the high affinity of complexes [V^IV^(L^1–4^)_2_] for BSA. Therefore, the group that seems to influence the most the strength of the interaction with BSA is the one connected to the sulfur atom: the replacement of CH_3_ by CH_2_–Ph increases twice the quenching constant. This interpretation is also supported by docking simulations.

**Table 6:** Binding and Fitting Parameters Obtained from the Fluorescence Titration Experiments, Measured at Room Temperature

complex	*K*_SV_ (M^–1^)	*r*^2^	*K*_BC_	*n*	*r*^2^
1	1.1 × 10^5^	0.995	2.6 × 10^4^	0.89	0.985
2	2.1 × 10^5^	0.997	4.0 × 10^4^	0.88	0.973
3	1.2 × 10^5^	0.996	1.8 × 10^4^	0.85	0.999
4	2.1 × 10^5^	0.997	3.6 × 10^4^	0.87	0.991

The double logarithm plot of log[(*I*_0_–*I*)/*I*] *vs* log[*Q*], where *Q* is the concentration of complexes **1**–**4** (see eq 2), is depicted in Figure S15 and allows us to obtain the binding constants *K*_BC_ (Table 6). If it is assumed that the values of *K*_BC_, in the range (1.8–4.0) × 10^4^, are correct, this would indicate a reversible binding of the metal complexes to BSA. It must be observed that, considering the existence in solution of [V^V^(L^1–4^)_2_]^+^ and [V^V^O_2_(L^1–4^)]^−^ besides [V^IV^(L^1–4^)_2_], as suggested by ESI-MS and DFT techniques, a mixture of these species may contribute to the affinity of **1**–**4** to BSA.

#### Study of BSA Interaction through Docking Analysis

**Figure 11:**
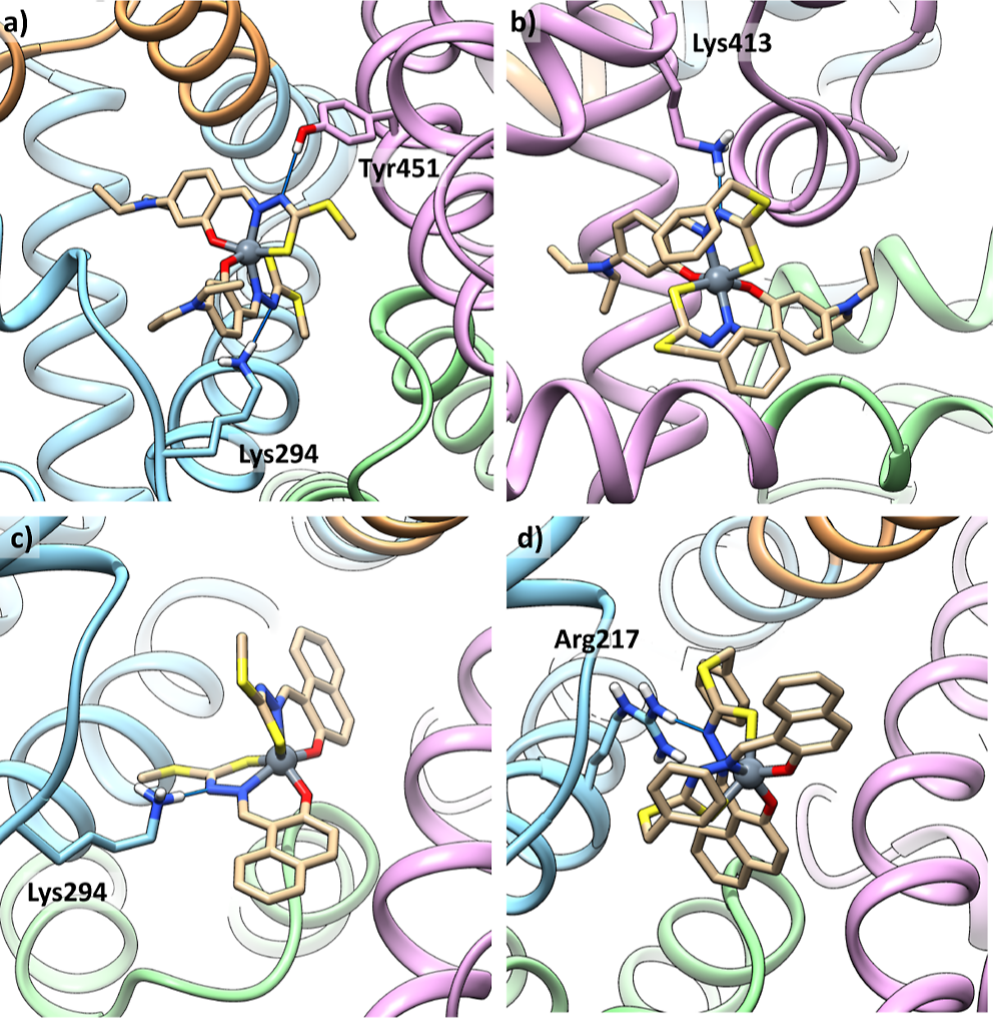
Best representative solutions of the most stable clusters for the interaction of [V^IV^(L^1–4^)_2_] with BSA: (a) *mer*-[V^IV^(L^1^)_2_] at the interface IIA/IIIA; (b) *mer*-[V^IV^(L^2^)_2_] at the interface IIB/IIIA; (c) *fac*-[V^IV^(L^3^)_2_] at the interface IIA/IIIA; and (d) *fac*-[V^IV^(L^4^)_2_] at the interface IIA/IIIA. Subdomains IIA, IIIA, IB, and IIB are depicted in cyan, purple, brown, and green, respectively. Interacting residues are also shown.

Dockings of the most stable isomers of [V^IV^(L^1–4^)_2_], derived from the SC-XRD structures and DFT calculations, toward BSA highlight several binding modes with scoring values (*F*_max_) ranging from 15.5 to 24.5 GoldScore units. Although the scoring values are similar indicating the absence of binding specificity, the affinity order *mer*-[V^IV^(L^2^)_2_] ∼ *fac*-[V^IV^(L^3^)_2_] > *mer*-[V^IV^(L^1^)_2_] ∼ *fac*-[V^IV^(L^4^)_2_] is predicted for this series of complexes. The best solutions are located at the interfaces of subdomains IIA/IIIA for [V^IV^(L^1,3,4^)_2_] (Figure 11a,c,d) and IIB/IIIA for *mer*-[V^IV^(L^2^)_2_] (Figure 11b). Additional solutions with slightly lower affinity are found at interface IA/IIA. All these interfacial regions are internal pockets reported to be common binding sites for ligands, including metal species.^146,147^ Notably, the adducts formed at these sites are close to Trp213, in line with the fluorescence quenching observed experimentally. Each adduct is stabilized inside the binding site by at least one hydrogen bond between the aza functionality of L^1–4^ with hydroxyl groups of Tyr or NH groups of Arg, Asn or Lys side chains (Table 7 and Tables S5–S8).

**Table 7:** Blind Docking Results for the Interaction of [V^IV^(L^1–4^)_2_] with Bovine Serum Albumin

complex	region	*F*_max_^a^	*F*_mean_^b^	interactions	pop^c^
[V^IV^(L^1^)_2_]	IIA/IIIA	20.8	17.9	NH_3_^+^–Lys294**···**NN; OH–Tyr451**···**NN’	43
[V^IV^(L^2^)_2_]	IIB/IIIA	24.5	20.3	NH_3_^+^–Lys413**···**NN	67
[V^IV^(L^3^)_2_]	IIA/IIIA	23.2	20.6	NH_3_^+^–Lys294**···**NN	63
[V^IV^(L^4^)_2_]	IIA/IIIA	19.4	14.7	NH_2_–Arg217**···**NN	17

a *Fitness* value for the most stable pose of each cluster (*F*_max_).

b Mean *Fitness* value of the GoldScore scoring function for each cluster (*F*_mean_).

c Number of solutions in the identified cluster.

**Figure 12:**
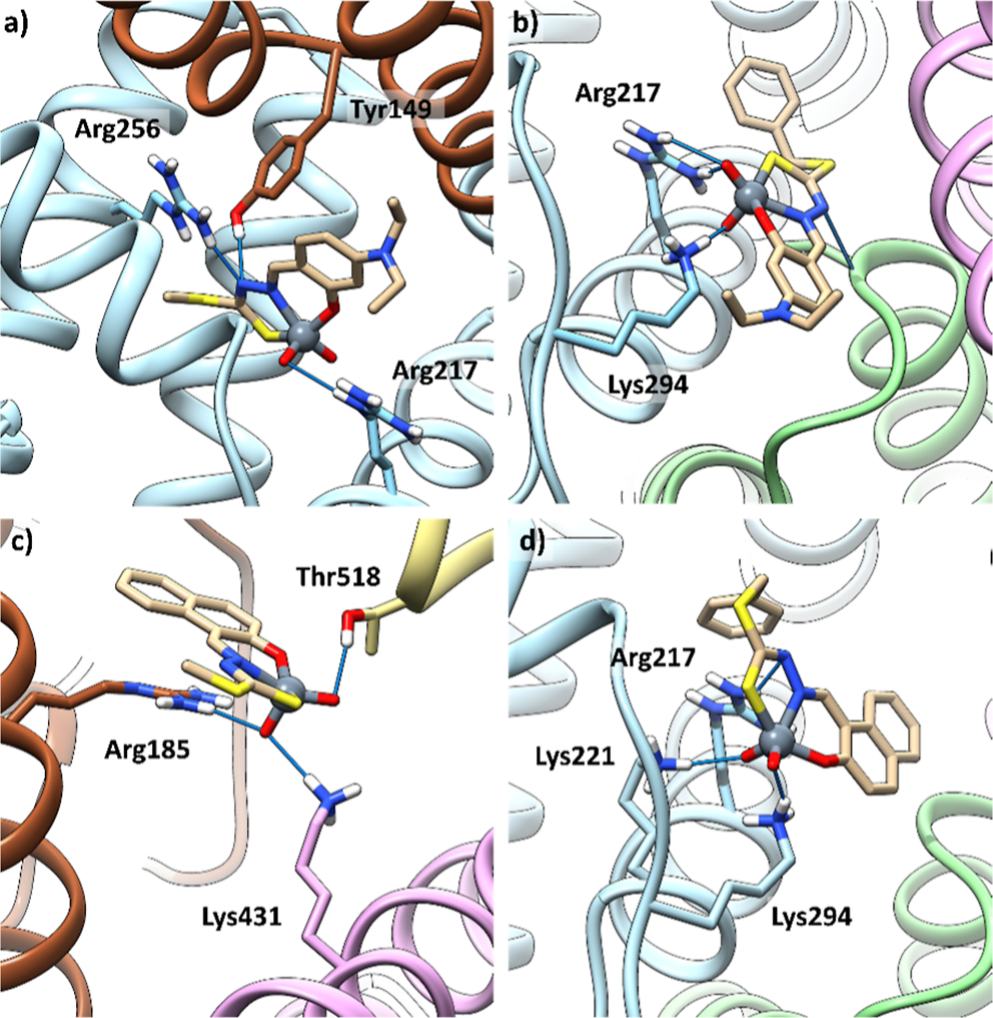
Best representative solutions of the most stable clusters for the interaction of [V^V^O_2_(L^1–4^)]^−^ with BSA: (a) [V^V^O_2_(L^1^)]^−^ at the interface IIA/IB; (b) [V^V^O_2_(L^2^)]^−^ at the interface IIA/IIB; (c) [V^V^O_2_(L^3^)]^−^ at the interface IB/IIIB; and (d) [V^V^O_2_(L^4^)]^−^ at the interface IIA/IIB. Subdomains IIA, IIIA, IB, IIB, and IIIB are depicted in cyan, purple, brown, green and tan, respectively. Interacting residues are also shown.

Docking results for [V^V^O_2_(L^1–4^)]^−^ show a similar trend, and the affinity order is [V^V^O_2_(L^2^)]^−^ ∼ [V^V^O_2_(L^1^)]^−^ > [V^V^O_2_(L^3^)]^−^ ∼ [V^V^O_2_(L^4^)]^−^, with the best solutions located at the interfaces of subdomains IIA/IB for [V^V^O_2_(L^1^)]^−^ (Figure 12a), IIA/IIB for [V^V^O_2_(L^2–4^)]^−^ (Figure 12b,d), and IB/IIIB for [V^V^O_2_(L^3^)]^−^ (Figure 12c). Also, for this series of complexes, additional solutions with slight lower affinity are found at interfaces IIIA/IB, IA/IIA, and IIA/IB. Similar to the non-oxido V^IV^ species, with [V^V^O_2_(L^1–4^)]^−^ hydrogen bonds are formed between the aza functionality of L^1–4^ and V^V^=O_oxido_ with hydroxyl groups of Tyr and Thr or NH groups of Arg, Asn, or Lys side chains (Table 8 and Tables S9–S12). For [V^V^O_2_(L^1–4^)]^−^ too, the simulated adducts are in quite proximity of residue Trp213.

**Table 8:** Blind Docking Results for the Interaction of [V^V^O_2_(L^1–4^)]^−^ with Bovine Serum Albumin

complex	region	*F*_max_^a^	*F*_mean_^b^	interactions	pop^c^
[V^V^O_2_(L^1^)]^−^	IIA/IB	22.8	21.4	NH_2_–Arg217**···**VO_eq_/NN; HO–Tyr149**···**NN	56
[V^V^O_2_(L^2^)]^−^	IIA/IIB	23.3	21.8	NH_3_^+^–Lys294**···**VO_ax_; NH_2_–Arg217**···**VO_ax_	4
[V^V^O_2_(L^3^)]^−^	IB/IIIB	21.3	17.6	NH_2_–Arg185/NH_3_^+^–Lys431**···**VO_ax_; OH–Thr518**···**VO_eq_	46
[V^V^O_2_(L^4^)]^−^	IIA/IIB	21.9	20.8	NH_3_^+^–Lys294**···**VO_eq_; NH_3_^+^–Lys221**···**VO_ax_; NH_2_–Arg217**···**NN	66

a *Fitness* value for the most stable pose of each cluster (*F*_max_).

b Mean *Fitness* value of the GoldScore scoring function for each cluster (*F*_mean_).

c Number of solutions in the identified cluster.

Concerning the oxidized species [V^V^(L^1–4^)_2_]^+^, solution analogues to those found for the [V^IV^(L^1–4^)_2_] are obtained. This is not surprising considering the high geometry similarity between the two series of complexes.

### *In Vitro* Cytotoxicity Studies

#### MTT Assay

**Figure 13:**
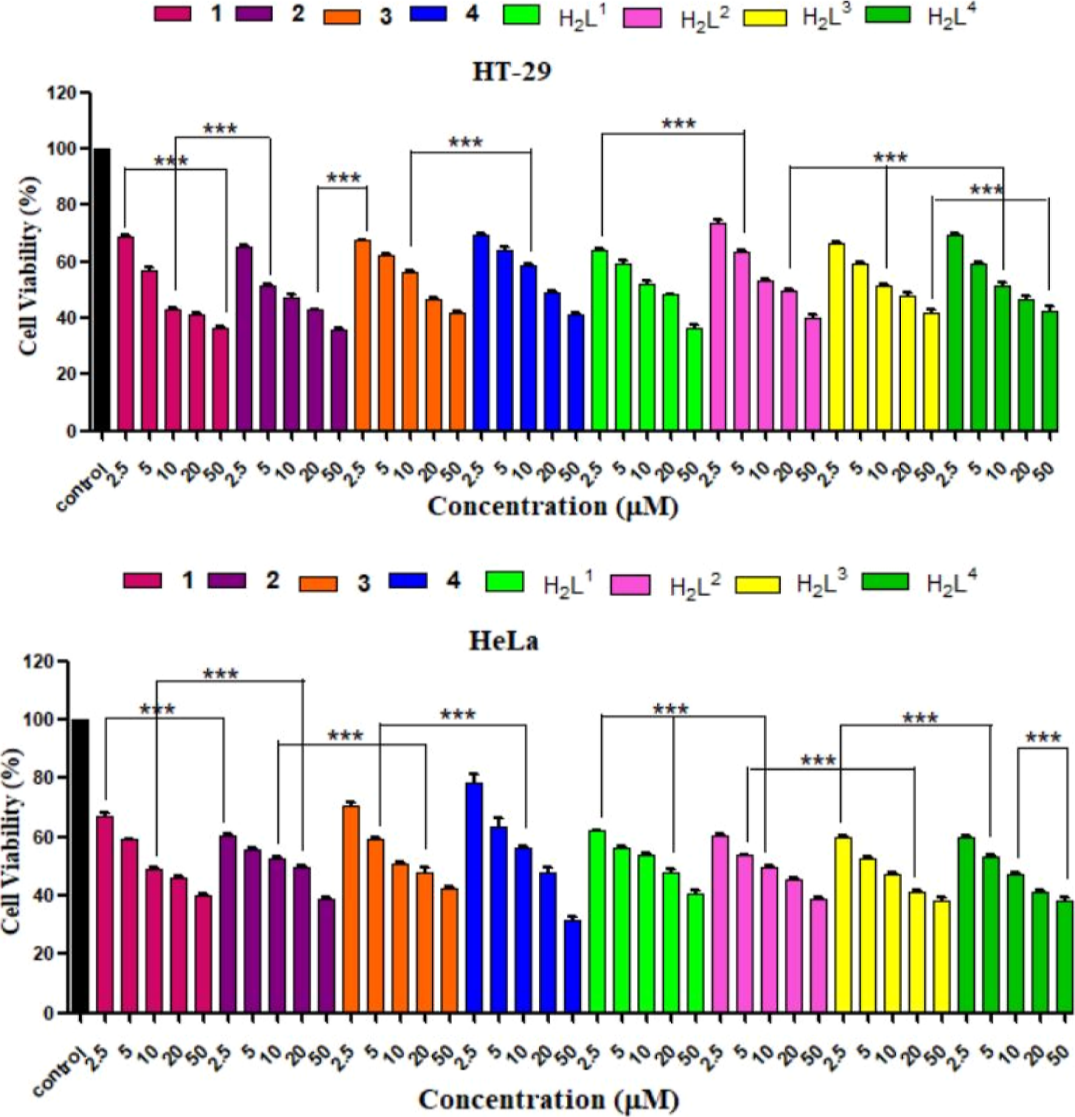
Cytotoxicity profiles of complexes **1**–**4** for HT-29 and HeLa cell lines after 48 h of incubation. The cells were treated with varying concentrations (2.5, 5, 10, 20, and 50 μM) of the tested complexes, and the cell viability was measured using the MTT assay. Data are reported as the mean ± SD for *n* = 4; the symbol *** represents a statistical significance of *p* < 0.001 as compared to the control.

The cytotoxicity potentials of dithiocarbazate ligand precursors (H_2_L^1–4^) and non-oxido vanadium(IV) complexes **1**–**4** were examined through MTT assay against two cancer (HT-29 and HeLa) and one normal (NIH-3T3) cell lines to check the specificity of the metal species toward cancer cells. From Table 9 and Figures 13 and S16, it can be observed that compounds having the −NEt_2_ group attached in the ligand backbone (**1** and **2**) are more active than the ligands having the −Ph group (**3** and **4**). Therefore, in both cell lines, the cytotoxicity potential of **1**–**4** follows the order **2 > 1 > 3 > 4**. The IC_50_ values of **1**–**4** are close in the two cancer cell lines, suggesting that these compounds do not show cell type-dependent nature. From the data depicted in Table 9, we can make other important observations, namely, that in cancer cells compounds **1** and **2** (derived from the ligands containing the −NEt_2_ group) are more active than their corresponding free ligands (H_2_L^1–2^), while the free ligands containing the −Ph group (H_2_L^3–4^) are more cytotoxic than their respective vanadium species (**3** and **4**). In other words, in the case of **3** and **4**, complexation does not improve the cytotoxic activity like for **1** and **2**.

**Table 9:** IC_50_ Values (μM) Calculated for the Ligand Precursors and Corresponding Vanadium Complexes Against the HT-29, HeLa, and NIH-3T3 Cells Upon 48 h of Exposure^a^

complex	HT-29	selectivity index (SI)	HeLa	selectivity index (SI)	NIH-3T3
H_2_L^1^	14.7 ± 0.6	1.6	15.7 ± 1.9	1.5	23.5 ± 0.6
**1**	7.2 ± 0.6	1.2	9.3 ± 0.2	0.9	8.7 ± 1.1
H_2_L^2^	17.9 ± 0.8	1.3	16.5 ± 1.4	1.4	23.1 ± 2.3
**2**	6.1 ± 2.0	2.2	8.2 ± 0.2	1.6	13.3 ± 1.3
H_2_L^3^	12.6 ± 2.1	>4	6.3 ± 0.1	>8	>50
**3**	16.2 ± 0.4	0.6	11.1 ± 0.9	0.9	10.3 ± 2.2
H_2_L^4^	13.1 ± 3.2	3.7	7.5 ± 0.3	6.6	49.2 ± 1.6
**4**	18.7 ± 1.6	0.6	17.1 ± 0.1	0.7	11.4 ± 3.1
cisplatin^b^	32.7 ± 0.6		25.5 ± 0.8		

a Results are the mean ± SD of two independent experiments done with four replicates.

b Data taken from refs (79) and (148).

Additionally, it is found that compounds **1**–**4** are more cytotoxic toward the normal cells than the ligand precursors. This accounts for the selectivity index (SI value). Among the complexes, **2** is more selective toward both cancer cells as compared to its ligand precursor, H_2_L^2^ (Table 9). These results provide the clear idea that compound **2**, having high cytotoxicity and better SI, is the most promising complex of the series. Also, BSA binding studies, obtained through experimental and DFT methods, are in line with the cytotoxicity results, indicating that **2** with higher protein binding affinity also shows higher cytotoxicity potential.

Another interesting thing observed is the direct relationship which apparently exists between the IC_50_ values and the gap (mV) between the oxidation and reduction potentials for the complexes **1**–**4**, *i.e.*, the lower the gap, the lower the IC_50_ values (Figure S17). The same trend is observed for the SI index.

It is also significant to discuss here that **1**–**4** show comparable or even better cytotoxic results than worldwide used chemotherapeutic drugs; for example, cisplatin under identical experimental conditions against HT-29 and HeLa cancer cell lines shows IC_50_ values of 32.7 ± 0.6 and 25.5 ± 0.8 μM, respectively.^79,148^ The present series of compounds also exhibits higher cytotoxicity than our previously reported non-oxido vanadium(IV) complexes with ONO donor ligands against cancer cell lines such as HeLa, A2780, and PC3.^64,65^ Additionally, the comparison between the cytotoxic activity of this new series of non-oxido vanadium(IV) complexes formed by ONS donors and other previously reported non-oxido V^IV^ species shows that **1**–**4** have analogous or even better cytotoxicity.^43,67^

In the light of ESI-MS and DFT results, which suggested that, besides [V^IV^(L^1–4^)_2_], the oxidation products [V^V^(L^1–4^)_2_]^+^ and [V^V^O_2_(L^1–4^)]^−^ exist in solution, this mixture of species could be responsible for the cytotoxic activity of **1**–**4**. As the formation of a small amount of [V^V^O_2_(L^1–4^)]^−^ implies the presence of an equivalent amount of free ligands in solution, cytotoxicity may also have the contribution of H_2_L^1–4^. However, because all IC_50_ values are in a rather narrow range (*ca.* 6–19 μM) and the amounts of free ligands formed are quite small, we expect the contribution of H_2_L^1–4^ to the cytotoxicity to be small.

#### Nuclear Staining Assay

**Figure 14:**
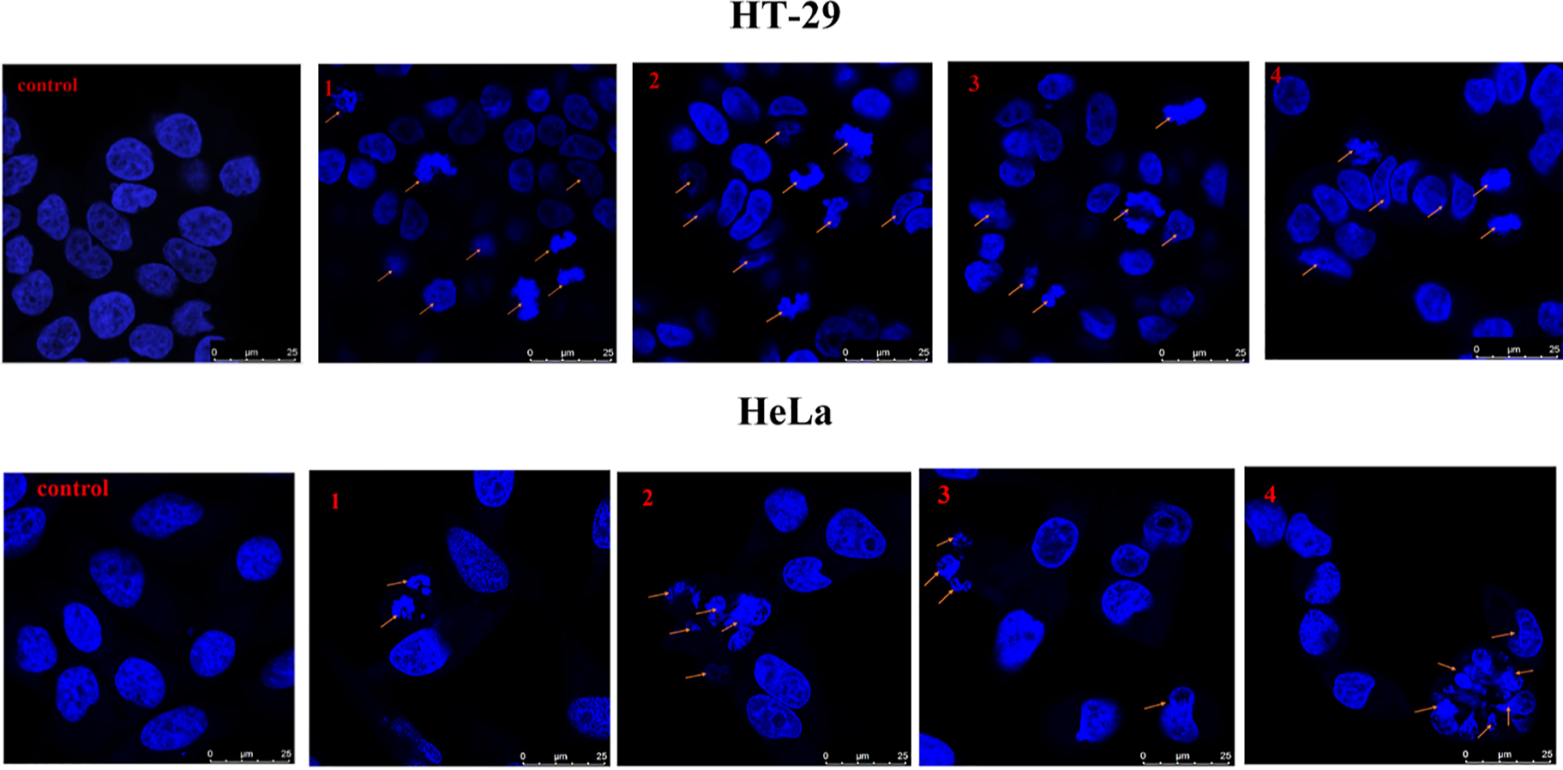
Morphologies of HT-29 (upper panel) and HeLa (lower panel) cells treated with complexes **1–4** at a concentration of 10 μM for 24 h. The cells were visualized under a confocal microscope after staining with DAPI (blue color, nucleus). The scale bar corresponds to 25 μm.

Apoptosis is normally considered as the “cleanest” pathway of cell death since the cellular contents do not leak; moreover, no inflammation takes place and the dead cells may be cleanly eliminated by the immune system. DAPI, a nuclear staining agent, is helpful to localize any nuclear alterations in cells, which further helps in the study of the mechanism of cell death in response to the treatment with the vanadium complexes. The DAPI staining images of **1**–**4** against HeLa and HT-29 cell lines are depicted in Figure 14. From the figure, the change in the nuclei morphology is clearly observed in the presence of the complexes, as compared to the control cell image. In the treated HT-29 cells, shrinking of the cells, fragmented, and condensed chromatin bodies are observed, while in HeLa cells—in addition to these nuclear changes—nuclear blebbing is also revealed after the treatment with **1**–**4**. The observed nuclear changes are the indication of apoptotic cell death induced by the complexes. Also, in the case of HeLa, a decrease in the number of cells is noticed after the treatment with the metal drugs as compared to the control. Therefore, from all these observations, we can state that programmed cell death (apoptosis)^149,150^ occurs in cancer cells in response to the treatment with the synthesized vanadium drugs.

#### Apoptosis Assay

**Figure 15:**
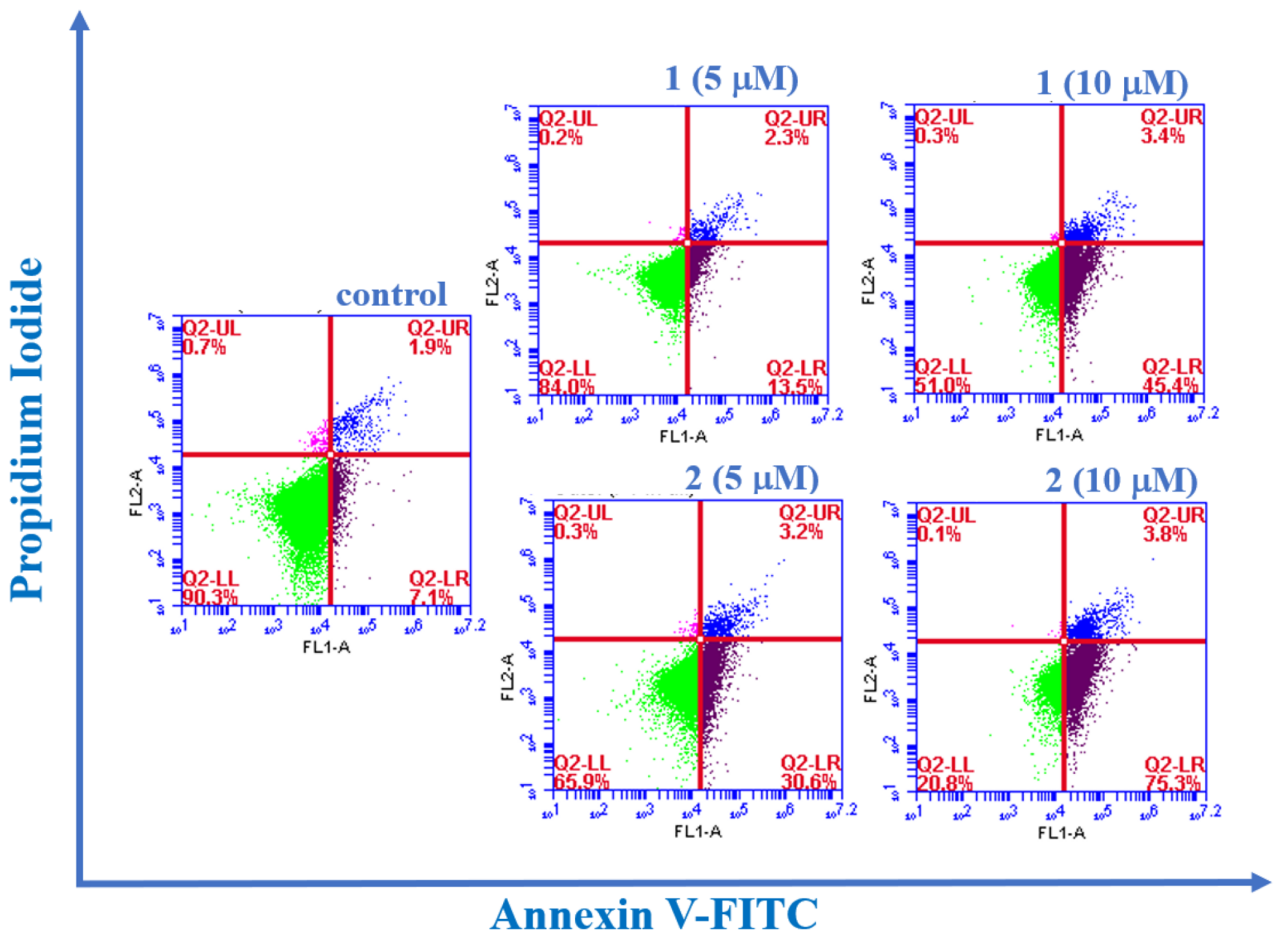
Quadrant graphs of cell apoptosis analysis in HT-29 cells treated with 5 and 10 μM concentrations of **1** and **2** for 48 h incubation time. Control indicates the untreated group.

From the DAPI staining assay, we got the indication that the apoptosis mode of cell death has occurred in cancer cells (HT-29 and HeLa) after the treatment with **1**–**4**. Therefore, to further confirm this and to determine the amount of apoptosis, annexin V FITC and PI double staining assay were performed in the HT-29 cell line on complexes **1** and **2** as representative examples. From Figure 15, it emerges that the sum of Q2(LR) and Q2(UR) regions is increased in both complexes as compared to the control; these two regions stand for early and late apoptosis cell percentages, respectively. The results suggest that the apoptotic amount is increased after the treatment with **1**–**4** and increases with the complex concentration from 5 to 10 μM. In particular, with 5 μM concentration, after the treatment with **1** and **2**, the apoptotic cells are 15.8 and 33.8%, and with 10 μM, they are 48.8 and 79.1%, while only 9% was noticed in the control. Hence, the overall result indicates that concentration-dependent apoptotic cell death occurs in HT-29 cells upon being treated with **1** and **2**.

## Conclusions

In its most stable higher oxidation states, +IV and +V, vanadium forms many types of compounds containing V=O bonds, *e.g.*, V^IV^O^2+^-, V^V^O^3+^-, and V^V^O_2_^+^-based complexes. In contrast, only very few non-oxido V^IV^ or V^V^ complexes have been isolated and structurally characterized. So, for these non-oxido complexes to be stable in the solid state or in water-containing solvents, preventing hydrolysis to form V-oxido species, the ligands must bind the V center quite strongly.

In this work, we report the preparation and characterization in the solid state and in solution of a group of new mononuclear non-oxido V^IV^ complexes, [V^IV^(L^1–4^)_2_] (**1**–**4**), the ligands being tridentate ONS chelating S-alkyl/aryl-substituted dithiocarbazate compounds. Three of these complexes, [V^IV^(L^1–3^)_2_] (**1**–**3**), were analyzed by single-crystal X-ray diffraction studies, revealing that the coordination geometry around the V center is distorted octahedral (**1** and **2**) or trigonal prismatic (**3**). Additionally, considering the formed O_2_N_2_S_2_ coordination environment around the V^IV^ center, **1** and **2** correspond to the *mer* isomers, while in **3,** a *fac* arrangement is formed. Interestingly, EPR data and DFT calculations indicate the coexistence of *mer* and *fac* isomers in solution, with comparable stability; probably, subtle factors like the solubility and/or the crystal packing may determine the isolation of one of the two isomeric forms in the solid state. To the best of our knowledge, this is one of the first studies in which the *mer* and *fac* isomers of non-oxido V^IV^ species are in equilibrium in solution, and this possibility should be considered in the future for the characterization of similar compounds.

It is worth noting that EPR, UV–vis, and ESI-MS data allowed to confirm that the obtained [V^IV^(L^1–4^)_2_] complexes are quite stable in solution for several hours, but suggested their partial oxidation to [V^V^(L^1–4^)_2_]^+^ and [V^V^O_2_(L^1–4^)]^−^, these changes proceeding faster as the relative amount of water and time increase.

Since we focus on the use vanadium compounds as pharmaceuticals, namely, for cancer treatment, the understanding of the interaction of complexes [V^IV^(L^1–4^)_2_] with proteins is relevant; this was studied with BSA by fluorescence quenching and computational methods. Docking calculations revealed non-covalent interactions with different regions of BSA, particularly with Tyr, Lys, Arg, Asn, and Thr residues. On the one hand, they allowed to confirm the fluorescence quenching results, since the adducts formed are found close to Trp213 residue; on the other hand, they highlighted that [V^IV^(L^1–4^)_2_], [V^V^(L^1–4^)_2_]^+^, and [V^V^O_2_(L^1–4^)]^−^ interact with different strengths and different subdomains of the protein, depending on the vanadium species and on the ligand coordinated.

Concerning their possible use as anti-cancer agents, *in vitro* cytotoxicity of all the complexes (and free ligands for comparison) was assayed against HT-29 and HeLa cancer cell lines and compared with NIH-3T3 normal cells. The results suggested that all four complexes are cytotoxic and induce cell death by apoptosis. However, although the compounds were normally more active against the cancer cells than in the normal cells, the selectivity is not high. Concerning the identity of the active species triggering apoptosis, we highlight that, despite the considerable stability of the complexes [V^IV^(L^1–4^)_2_], oxidation/hydrolysis certainly takes place to some extent during the experiments in water. Therefore, a mixture of V^IV^, V^V^, and V^V^O_2_ species, being present in these systems, could be responsible for the activity observed. The small amounts of free ligands formed may also contribute. These results reinforce our recent conclusions that, regardless of the oxidation state of one of these administered vanadium drugs, a mixture of oxido and non-oxido V^IV^ and V^V^ species could be formed at the physiological conditions, their relative amount depending on the nature of the ligand and thermodynamic stability of the initial compound; therefore, the biological action should be interpreted and explained assuming the existence of more than one V species in solution, probably in the +IV and +V oxidation states.

## Supporting Information Available

Tables with crystal data and refinement details for the X-ray structure determinations, with calculated structural details for [V^IV^(L^1–4^)_2_], [V^V^O_2_(L^1–4^)]^−^, and [V^V^(L^1–4^)_2_]^+^ complexes, docking results for the interaction of [V^IV^(L^1–4^)_2_] and [V^V^O_2_(L^1–4^)]^−^ with BSA, figures with crystallographic details, UV–vis spectra of complexes **1**–**4**, experimental and calculated ESI-MS spectra of **1**, cyclic voltammogram of H_2_L^4^, double logarithm plot of log[(*I*_0_–*I*)/*I*] *vs* log[*Q*] for **1**–**4**, cytotoxicity profiles of **1**–**4** for NIH-3T3 cell lines, and correlations between IC_50_ and gap between the oxidation and reduction potentials for **1**–**4** (PDF)
