# Supplementary material for: A Series of Non-Oxido VIV Complexes of Dibasic ONS Donor Ligands: Solution Stability, Chemical Transformations, Protein Interactions, and Antiproliferative Activity
Source: Inorg Chem. 2023 May 8;62(20):7932–53. doi: 10.1021/acs.inorgchem.3c00753 (PMC10367067; doi:10.1021/acs.inorgchem.3c00753)
Supplement: Supplementary file 1 — ic3c00753_si_001.pdf [file ic3c00753_si_001.pdf]

SUPPORTING INFORMATION

A series of non-oxido V^{IV} complexes of dibasic ONS donor ligands: Solution stability, chemical transformations, protein interactions, and antiproliferative activity

Atanu Banerjee,[†] Sushree Aradhana Patra,[†] Gurunath Sahu,[†] and Giuseppe Sciortino,[⊥] Federico Pisanu,[&] Eugenio Garribba*[&] M. Fernanda N.N. Carvalho,[‖] Isabel Correia,[‖] João Costa Pessoa*[‖] Hans Reuter[‡] and Rupam Dinda*[†]

[†] *Department of Chemistry, National Institute of Technology, Rourkela, 769008 Odisha, India. E-mail: rupamdinda@nitrkl.ac.in*

[⊥] *Institute of Chemical Research of Catalonia (ICIQ), The Barcelona Institute of Science and Technology, 43007 Tarragona, Spain.*

[&] *Dipartimento di Medicina, Chirurgia e Farmacia, Università di Sassari, Viale San Pietro, I-07100 Sassari, Italy. E-mail: garribba@uniss.it*

[‖] *Centro de Química Estrutural and Departamento de Engenharia Química, Institute of Molecular Sciences, Instituto Superior Técnico, Universidade de Lisboa, Avenida Rovisco Pais, 1049-001 Lisboa, Portugal. E-mail: joao.pessoa@ist.utl.pt*

[‡] *Institute of Chemistry of New Materials, University of Osnabrück, Barbarastraße 6, 49069 Osnabruck, Germany.*

Table S1. Crystal data and refinement details for the X-ray structure determinations of the complexes **1−3**.

Complex	**1**	**2**	**3**
empirical formula	$C_{26}H_{34}N_6O_2S_4V$	$C_{38}H_{42}N_6O_2S_4V$	$C_{26}H_{20}N_4O_2S_4V$
formula weight/g·mol^{-1}	641.77	793.95	599.64
T/°C	100(2) K	296(2) K	296(2) K
crystal system	monoclinic	Triclinic	monoclinic
space group	*C*2/*c*	*P-1*	*P2/n*
a/ Å	24.0122(7)	12.9359(6)	8.7352(5)
b/ Å	9.2512(3)	13.2138(6)	6.4724(4)
c/ Å	13.5143(4)	13.6836(7)	22.3988(8)
α/°	90	111.236(2)°	90
β/°	107.905(2)°	109.701(2)°	90.163(2)°
γ/°	90	98.423(2)°	90
V/Å^3	2856.7(2)	1954.0(2)	1266.4(1)
Z, Z′	4, ½	2, 1	2, ½
ρ/g·cm^{-3}	1.492	1.349	1.573
µ/mm^{-1}	0.676	0.509	0.755
F(000)	1340	830	614
$2\Theta_{max}$	50°	50°	50°
reflections collected	57174	99011	33673
reflections unique/R_{int}	2508/0.0458	6874/0.0506	2235/0.0770
data/restraints/parameter	2508 / 0 / 183	6874 / 0 / 470	2235 / 0 / 171
Goodness-of-fit on F^2	1.054	1.036	1.050
R/wR_2 [I>2σ(I)]	0.0326/0.0793	0.0377/0.0914	0.0386/0.0866
R/wR_2 [all data]	0.0373/0.0827	0.0506/0.1002	0.0579/0.0961
largest diff. peak, hole/e·Å^{-3}	0.717 and -0.190	0.383 and -0.182	0.307 and -0.209
CCDC No.	2004341	2004342	2004343

Table S2. Selected bond lengths (Å) and bond angles (°) for calculated and experimental structures of $[V^{IV}(L^{1-4})_2]$.[a]

	**1**			**2**			**3**			**4**		
Bond/angle	*fac*	*fac'*	*mer*	*fac*	*fac'*	*mer*	*fac*	*fac'*	*mer*	*fac*	*fac'*	*mer*
$V–O_1$	1.877	1.874	1.895 (1.923)	1.877	1.875	1.895 (1.926)	1.882 (1.894)	1.875	1.833	1.882	1.875	1.833
$V–N_1$	2.109	2.105	2.079 (2.047)	2.109	2.105	2.077 (2.071)	2.110 (2.105)	2.097	2.069	2.110	2.097	2.070
$V–S_1$	2.399	2.379	2.379 (2.352)	2.400	2.380	2.378 (2.343)	2.382 (2.352)	2.368	2.438	2.382	2.368	2.438
$V–O_2$	1.877	1.880	1.895 (1.923)	1.877	1.881	1.896 (1.936)	1.882 (1.894)	1.887	1.833	1.882	1.887	1.833
$V–N_2$	2.109	2.090	2.079 (2.047)	2.109	2.090	2.079 (2.064)	2.110 (2.105)	2.087	2.069	2.110	2.088	2.070
$V–S_2$	2.399	2.398	2.379 (2.352)	2.400	2.398	2.381 (2.350)	2.382 (2.352)	2.385	2.438	2.382	2.386	2.438
$N_1–V–N_2$	118.8	121.4	153.9 (155.1)	118.8	122.0	154.0 (148.0)	122.9 (125.2)	121.1	161.7	122.7	121.2	161.2
$O_1–V–S_1$	124.6	123.1	156.3 (155.6)	124.6	123.4	156.4 (148.9)	124.8 (125.2)	122.0	164.0	124.7	122.0	163.4
$O_2–V–S_2$	124.6	120.6	156.3 (155.6)	124.6	120.9	156.6 (149.5)	124.8 (125.2)	119.7	164.0	124.7	119.8	163.4

[a] The experimental values extracted from X-ray structures are in parentheses.

Table S3. Selected bond lengths (Å) and bond angles (°) for calculated structures of $[V^{V}O_2(L^{1-4})]^-$.

Bond/angle	**1**	**2**	**3**	**4**
$V=O_1$	1.610	1.610	1.609	1.609
$V=O_2$	1.622	1.622	1.621	1.621
$V–O_3$	1.929	1.927	1.935	1.934
$V–N_1$	2.247	2.246	2.235	2.234
$V–S_1$	2.458	2.461	2.448	2.450
$O_1=V=O_2$	109.4	109.4	109.5	109.5
$O_1=V–O_3$	105.8	105.9	105.7	105.8
$O_1=V–N_1$	99.4	99.5	99.8	99.8
$O_1=V–S_1$	105.5	105.4	106.0	106.0

Table S4. Selected bond lengths (Å) and bond angles (°) for calculated structures of most stable isomers of $[V^{V}(L^{1-4})_2]^+$.

Bond/angle	**1** (*mer*)	**2** (*mer*)	**3** (*fac*)	**4** (*fac*)
$V–O_1$	1.810	1.810	1.782	1.783
$V–N_1$	2.056	2.056	2.095	2.096
$V–S_1$	2.350	2.349	2.350	2.349
$V–O_2$	1.810	1.812	1.782	1.783
$V–N_2$	2.056	2.059	2.095	2.096
$V–S_2$	2.350	2.345	2.350	2.349
$N_1–V–N_2$	156.0	155.8	95.7	95.8
$O_1–V–S_1$	161.6	160.5	113.8	113.9
$O_2–V–S_2$	161.6	162.2	113.8	113.9

Table S5. Blind docking results for the interaction of $[V^{IV}(L^1)_2]$ with bovine serum albumin.

Region	F_{max}[a]	F_{mean}[b]	Interactions	Pop.[c]	Rank
IIA/IIIA	20.8	17.9	NH_3^+–Lys294···NN; OH–Tyr451···NN’	43	I
IIB/IIIA	19.6	17.9	NH_3^+–Lys413···NN	24	II
IA/IIA	16.7	15.0	NH_3^+–Lys239···NN; NH_3^+–Lys242···NN	28	III

[a] *Fitness* value for the most stable pose of each cluster (F_{max}). [b] Mean *Fitness* value of the GoldScore scoring function for each cluster (F_{mean}). [c] Number of solutions in the identified cluster.

Table S6. Blind docking results for the interaction of $[V^{IV}(L^2)_2]$ with bovine serum albumin.

Region	F_{max}[a]	F_{mean}[b]	Interactions	Pop.[c]	Rank
IIB/IIIA	24.5	20.3	NH_3^+–Lys413···NN	67	I
IIA/IIIA	20.0	15.4	NH_2–Arg217···NN; HO–Tyr451···NN’	51	II
IA/IIA	18.3	15.2	NH_3^+–Lys239···NN	4	III

[a] *Fitness* value for the most stable pose of each cluster (F_{max}). [b] Mean *Fitness* value of the GoldScore scoring function for each cluster (F_{mean}). [c] Number of solutions in the identified cluster.

Table S7. Blind docking results for the interaction of $[V^{IV}(L^3)_2]$ with bovine serum albumin.

Region	F_{max}[a]	F_{mean}[b]	Interactions	Pop.[c]	Rank
IIA/IIIA	23.2	20.6	NH_3^+–Lys294···NN	63	I
IIA/IIIB	15.5	14.3	NH_2–Arg427···NN	97	II
IA/IIA	15.8	15.3	NH_3^+–Lys242···NN	82	III

[a] *Fitness* value for the most stable pose of each cluster (F_{max}). [b] Mean *Fitness* value of the GoldScore scoring function for each cluster (F_{mean}). [c] Number of solutions in the identified cluster.

Table S8. Blind docking results for the interaction of $[V^{IV}(L^4)_2]$ with bovine serum albumin.

Region	F_{max}[a]	F_{mean}[b]	Interactions	Pop.[c]	Rank
IIA/IIIA	19.4	14.7	NH_2–Arg217···NN	17	I
IIA/IIIA	19.14	17.46	NH_3^+–Lys413···NN	11	II
IA/IIA	16.51	16.12	NH_3^+–Lys242···NN	6	III

[a] *Fitness* value for the most stable pose of each cluster (F_{max}). [b] Mean *Fitness* value of the GoldScore scoring function for each cluster (F_{mean}). [c] Number of solutions in the identified cluster.

Table S9. Blind docking results for the interaction of $[V^{V}O_2(L^1)]^-$ with bovine serum albumin.

Region	F_{max}[a]	F_{mean}[b]	Interactions	Pop.[c]	
IIA/IB	22.8	21.4	NH_2–Arg217···VO_{eq} / NN; HO–Tyr149···NN	56	I
IIA/IIIB	20.7	18.1	NH_2–Arg427···VO_{eq}; NH_2–Arg185···VO_{ax}	53	II
IIA/IIIA	19.4	18.0	NH_3^+–Lys249···VO_{ax}; NH_2–Arg217···NN	16	III
IIIA	17.9	13.6	NH_3^+–Lys413···VO; NH_2–Asn340···NN	43	IV
IA/IIA	17.0	16.1	NH_2–Arg10···VO_{eq}; OH–Ser65···NN	9	V

[a] *Fitness* value for the most stable pose of each cluster (F_{max}). [b] Mean *Fitness* value of the GoldScore scoring function for each cluster (F_{mean}). [c] Number of solutions in the identified cluster.

Table S10. Blind docking results for the interaction of $[V^VO_2(L^2)]^-$ with bovine serum albumin.

Region	F_{max}[a]	F_{mean}[b]	Interactions	Pop.[c]	Rank
IIA/IIB	23.3	21.8	NH_3^+–Lys294···VO_{ax}; NH_2–Arg217···VO_{ax}	4	I
IIIA/IB	21.8	18.7	NH_3^+–Lys431···VO_{ax}; NH_2–Arg427···VO_{eq}	46	II
IIA/IIB	21.3	18.9	NH_3^+–Lys294...VO_{eq}; NH_3^+–Lys221···VO_{ax}	4	III
IIIA	19.9	19.0	NH_2–Arg409···NN; OH–Ser488···VO_{ax}; OH–Tyr410···VO_{ax}	23	IV
IA/IIA	18.7	17.7	NH_2–Arg10···$VO_{ax/eq}$	24	V

[a] *Fitness* value for the most stable pose of each cluster (F_{max}). [b] Mean *Fitness* value of the GoldScore scoring function for each cluster (F_{mean}). [c] Number of solutions in the identified cluster.

Table S11. Blind docking results for the interaction of $[V^VO_2(L^3)]^-$ with bovine serum albumin.

Region	F_{max}[a]	F_{mean}[b]	Interactions	Pop.[c]	Rank
IB/IIIB	21.3	17.6	NH_2–Arg185 / NH_3^+–Lys431···VO_{ax}; OH–Thr518···VO_{eq}	46	I
IIA/IIB	19.7	18.4	NH_2–Arg217···NN; NH_3^+–Lys294···VO_{ax}	46	II
IA/IIA	19.1	18.0	NH_2–Arg10···$VO_{ax/eq}$	62	III
IIA/IB	19.1	18.4	NH_2–Arg256···NN; OH–Tyr149···NN	12	IV
IIIA	17.8	16.9	NH_2–Arg409···NN; NH_2–Asn390···NN; NH_3^+–Lys413···VO_{eq}	19	VII

[a] *Fitness* value for the most stable pose of each cluster (F_{max}). [b] Mean *Fitness* value of the GoldScore scoring function for each cluster (F_{mean}). [c] Number of solutions in the identified cluster.

Table S12. Blind docking results for the interaction of $[V^VO_2(L^4)]^-$ with bovine serum albumin.

Region	F_{max}[a]	F_{mean}[b]	Interactions	Pop.[c]	Rank
IIA/IIB	21.9	20.8	NH_3^+–Lys294···VO_{eq}; NH_3^+–Lys221···VO_{ax}; NH_2–Arg217···NN	66	I
IIIA/IB	20.9	14.9	NH_3^+–Lys431···VO_{ax}; NH_2–Arg185···VO_{ax}; OH–Thr518···VO_{eq}	34	II
IIIA	19.3	16.9	NH_3^+–Lys413···VO_{eq}; NH_2–Arg409···NN; NH_2–Asn390···NN	92	III
IA/IIA	18.8	17.8	NH_2–Arg10···$VO_{ax/eq}$	44	V

[a] *Fitness* value for the most stable pose of each cluster (F_{max}). [b] Mean *Fitness* value of the GoldScore scoring function for each cluster (F_{mean}). [c] Number of solutions in the identified cluster.

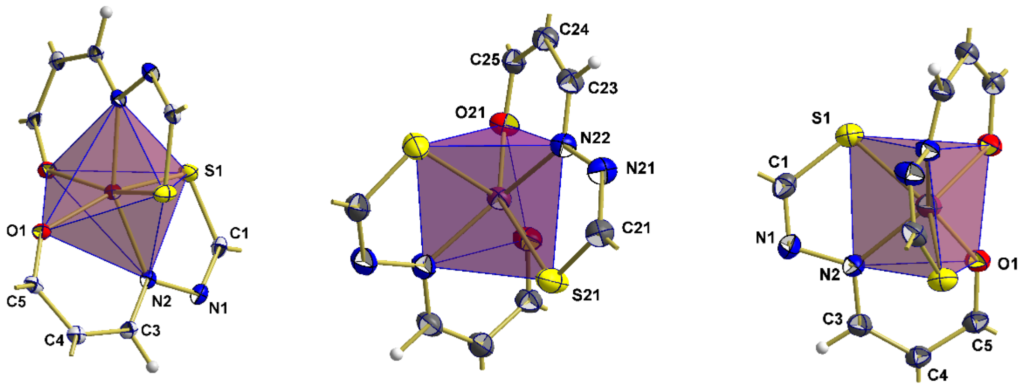

1 **2** **3**

Figure S1. Coordination geometries at the vanadium atoms of complexes **1** and **2** (distorted octahedral) and complex **3** (distorted trigonal prismatic).

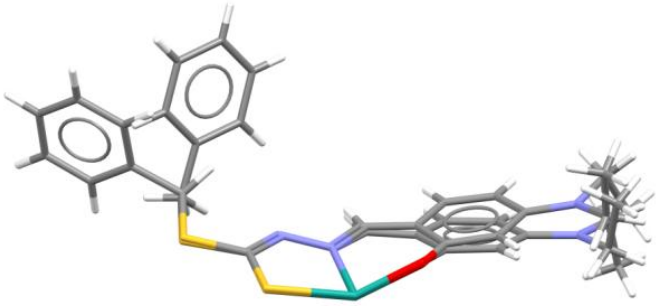

Figure S2. Overlay of the two crystallographic different ligands of **2** showing their different position.

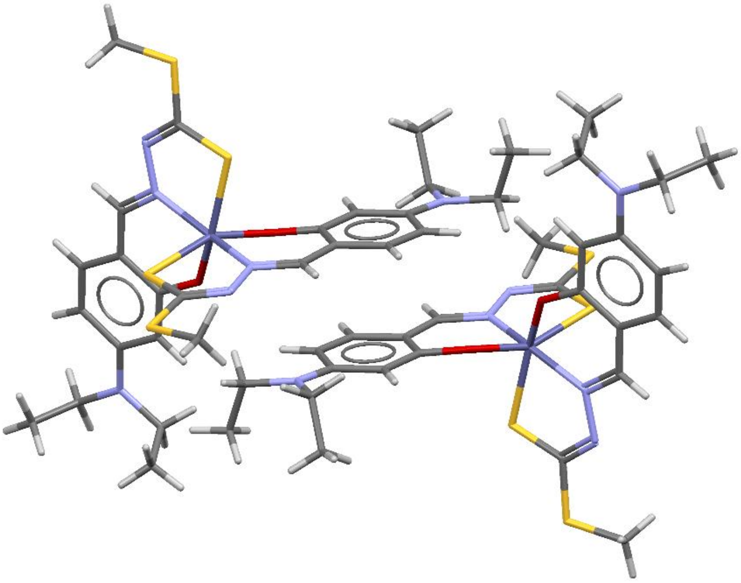

Figure S3. π-interaction between two complexes found in the crystal structure of **1**.

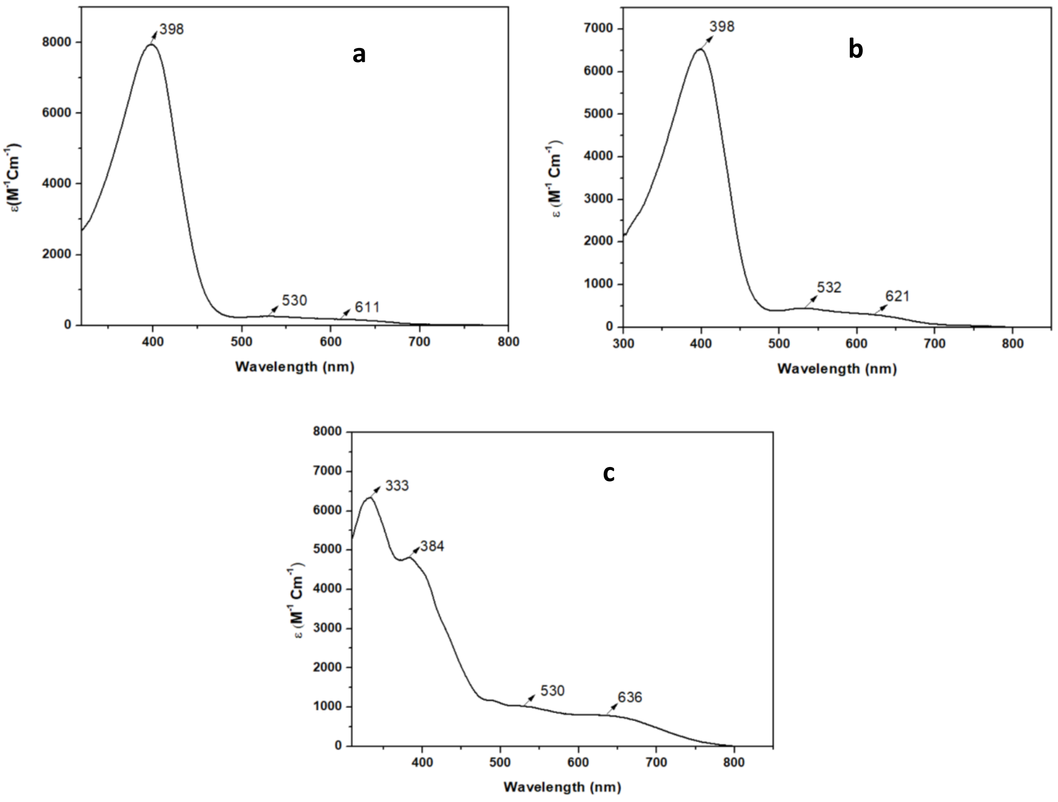

Figure S4. UV-Vis spectra of **1** (a), **2** (b), and **4** (c) in DMSO recorded with a concentration of 1.5 $\times$ 10^{-4} M and path length = 1 cm.

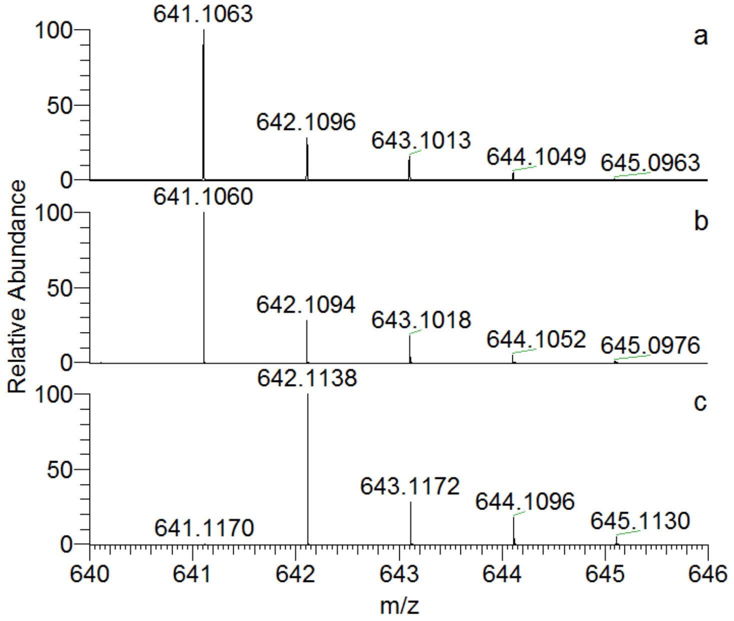

Figure S5. Region in the m/z range 640-646 of the ESI-MS(+) spectrum of complex **1** in CH_3CN (50 μM). (a) Experimental spectrum; (b) calculated isotopic pattern for $[V^{V}(L^{1})_2]^+$; (b) calculated isotopic pattern for $[V^{IV}(L^{1})_2]+H^+$.

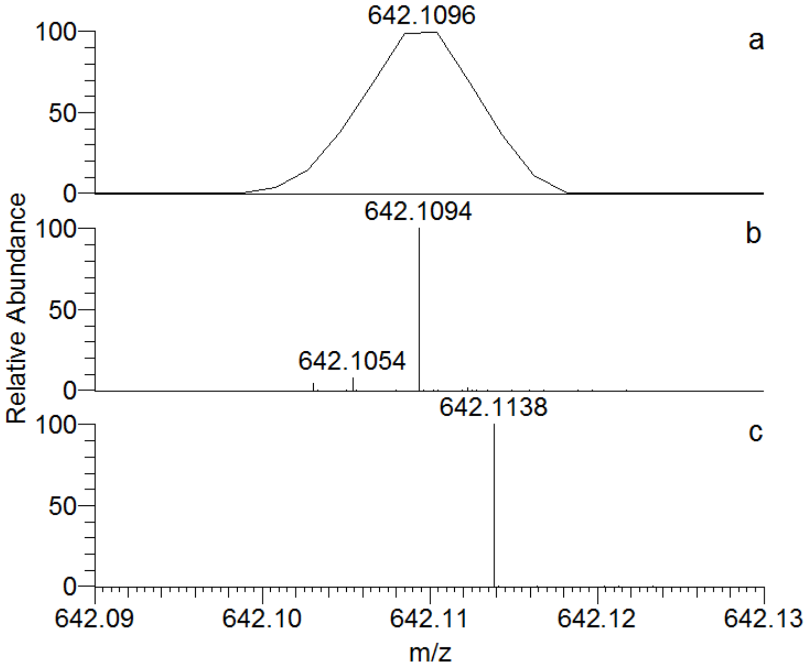

Figure S6. Region in the m/z range 642.09-642.13 of the ESI-MS(+) spectrum of complex **1** in CH_3CN (50 μM). (a) Experimental spectrum; (b) calculated isotopic pattern for $[V^{V}(L^{1})_2]^+$; (c) calculated isotopic pattern for $[V^{IV}(L^{1})_2]+H^+$.

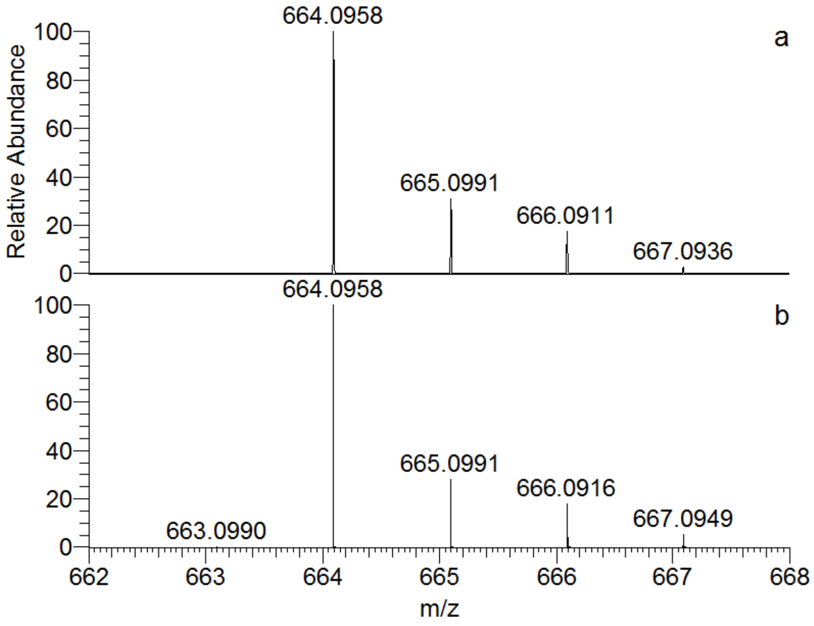

Figure S7. Region in the m/z range 662-668 of the ESI-MS(+) spectrum of complex **1** in CH_3CN (50 μM). (a) Experimental spectrum; (b) calculated isotopic pattern for $[V^{IV}(L^{1})_2]+Na^+$.

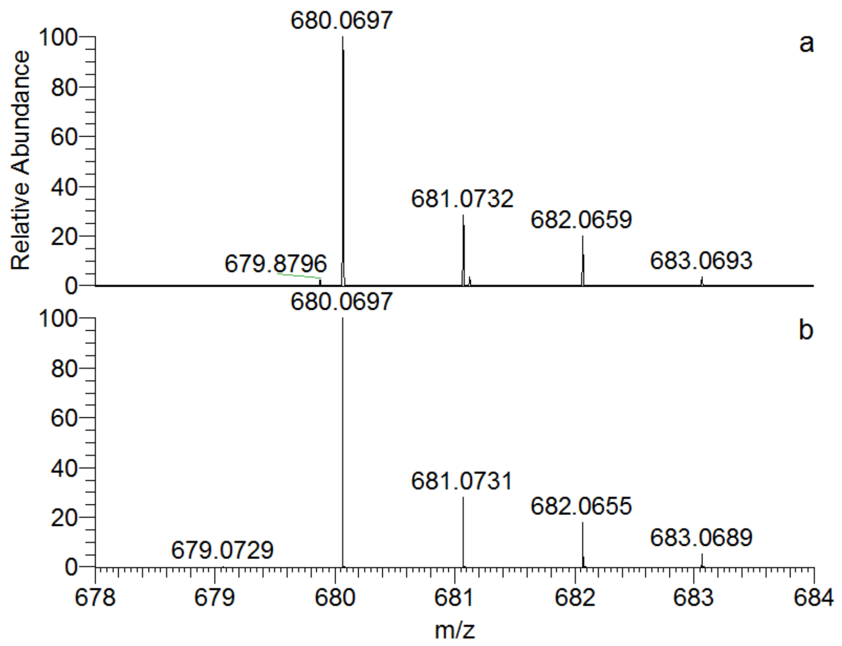

Figure S8. Region in the m/z range 678-684 of the ESI-MS(+) spectrum of complex **1** in CH_3CN (50 μM). (a) Experimental spectrum; (b) calculated isotopic pattern for $[V^{IV}(L^1)_2]+K^+$.

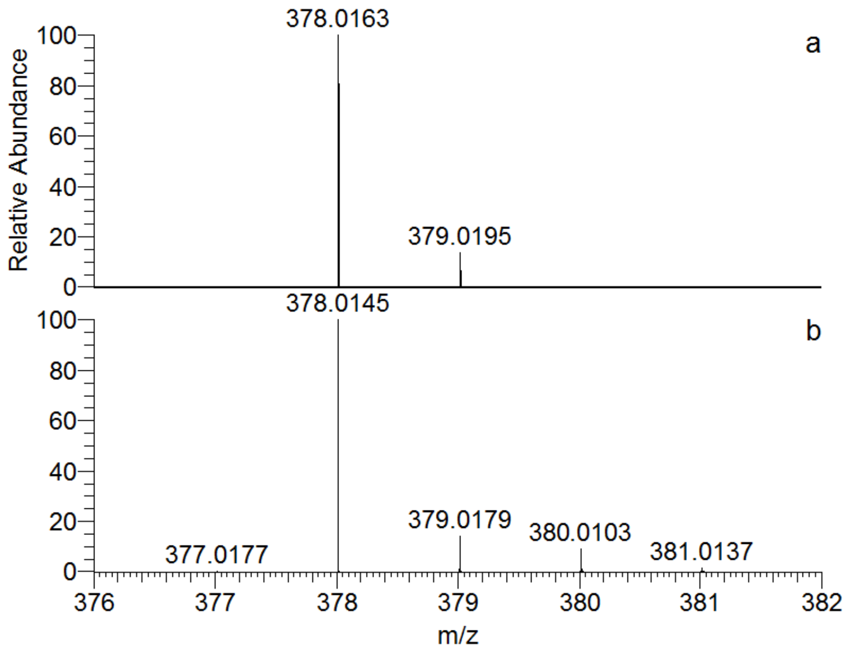

Figure S9. Region in the m/z range 376-382 of the ESI-MS(–) spectrum of complex **1** in CH_3CN (50 μM). (a) Experimental spectrum; (b) calculated isotopic pattern for $[V^{V}O_2(L^1)_2]^-$.

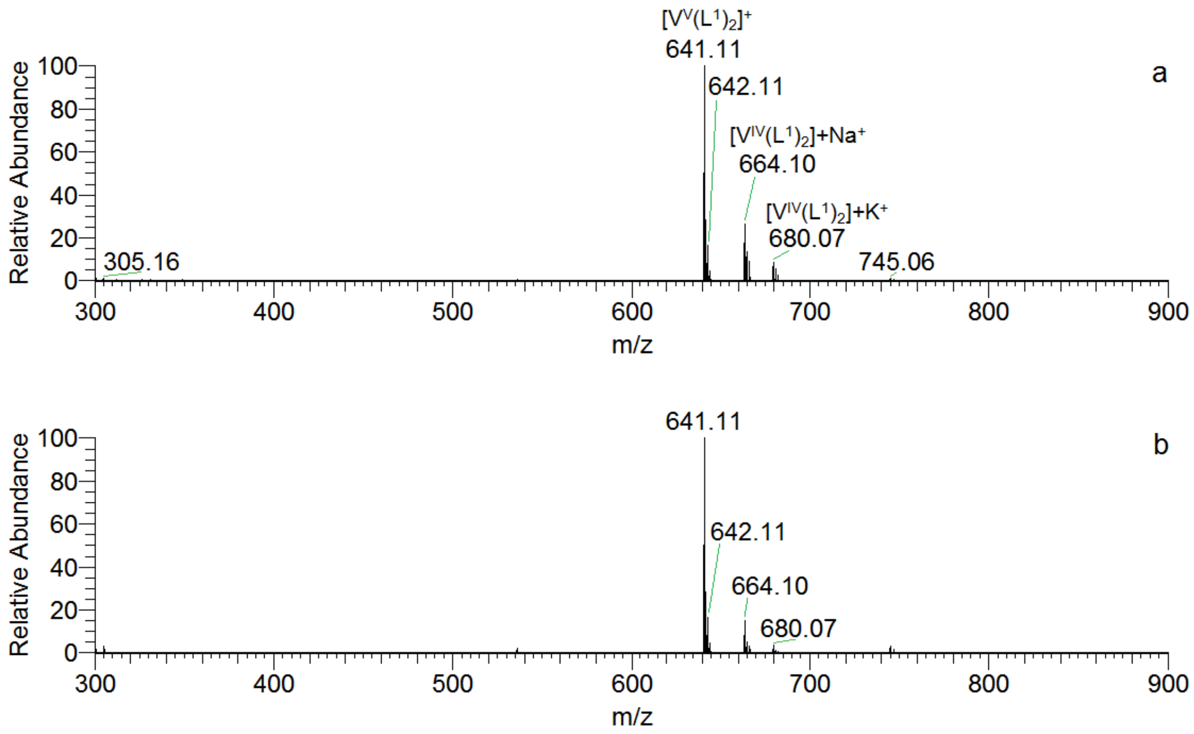

Figure S10. ESI-MS spectra in positive-ion mode recorded dissolving complex **1** in CH_3CN (50 μM) in CH_3CN (a) and $MeOH/H_2O$ 90/10 v/v (b).

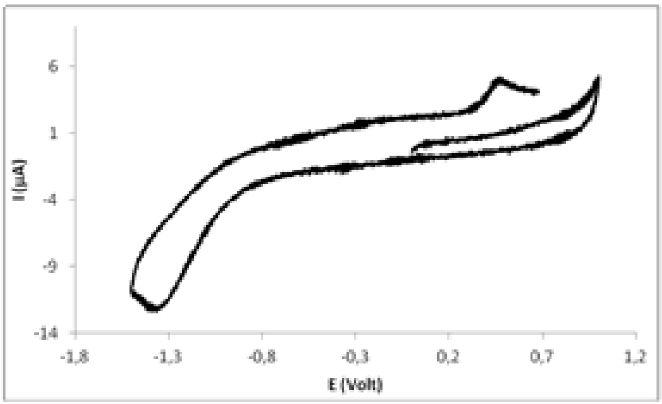

Figure S11. Cyclic voltammogram of H_2L^4 in DMSO at a scan rate of 200 mV s^{-1} obtained from Bu_4NBF_4 / CH_2Cl_2 (0.10 M).

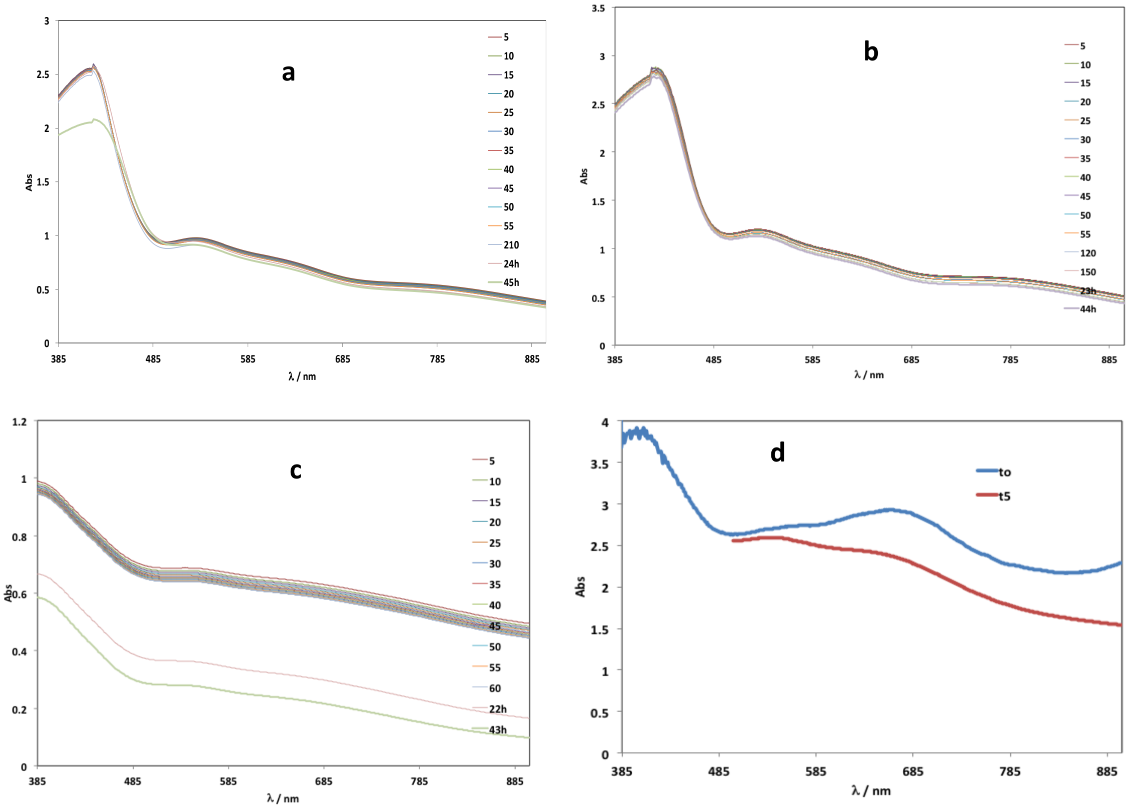

Figure S12. Time dependence of the UV-Vis absorption spectra of 10% DMSO/H_2O v/v solutions of: (a) complex **1** (1.66 × 10^{-4} M); (b) complex **2** (1.01 × 10^{-3} M); (c) complex **3** (1.03 × 10^{-3} M); (d) complex **4** (1.09 × 10^{-3} M). For **3** precipitations occurred with time, and for **4** it was immediate.

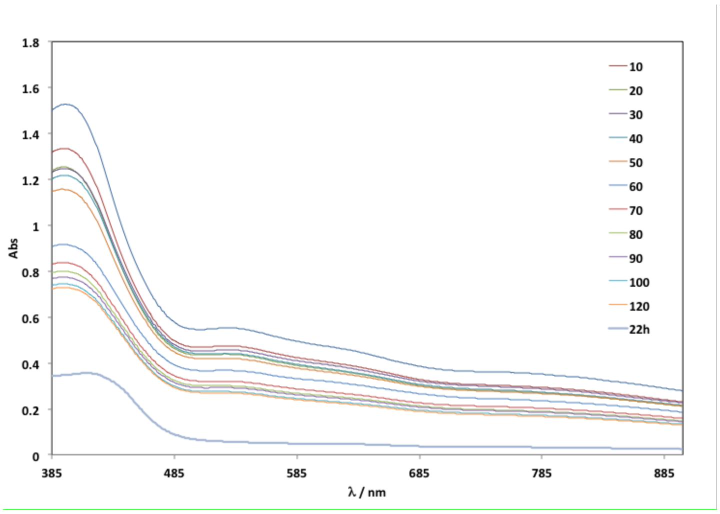

Figure S13. Time dependence of the UV-vis absorption spectra of 10% DMSO/MEM v/v solutions of **1** (9.97 × 10^{-4} M).

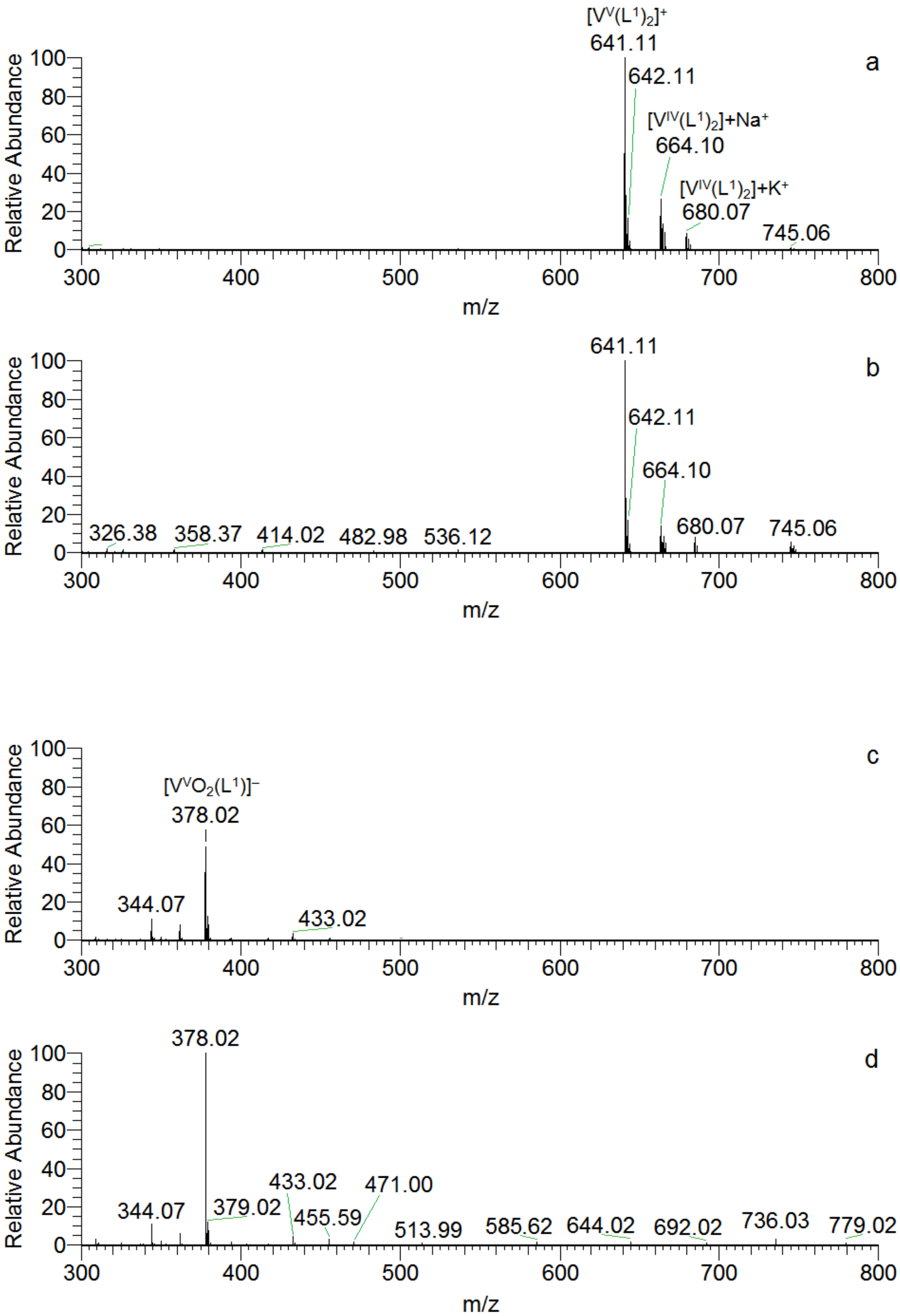

Figure S14. ESI-MS spectra in the positive-ion mode and negative-ion mode recorded dissolving complex **1** in MeOH/H_2O 90/10 v/v and diluting with CH_3CN. (a) ESI-MS(+) spectrum of **1** at t = 0 h; (b) ESI-MS(+) spectrum of **1** after t = 24 h; (c) ESI-MS(–) spectrum of **1** at t = 0; (d) ESI-MS(–) spectrum of **1** after t = 24 h.

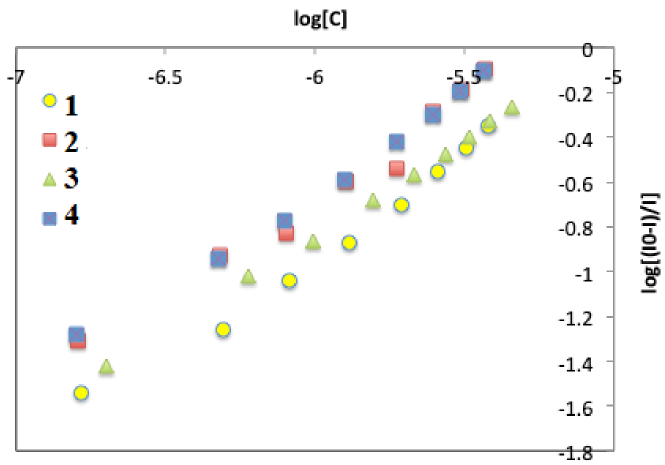

Figure S15. Double logarithm plot of log[(I_0-I)/I] *vs.* log[*Q*], where *Q* is the concentration of the quencher (i.e., one of complexes **1**–**4**).

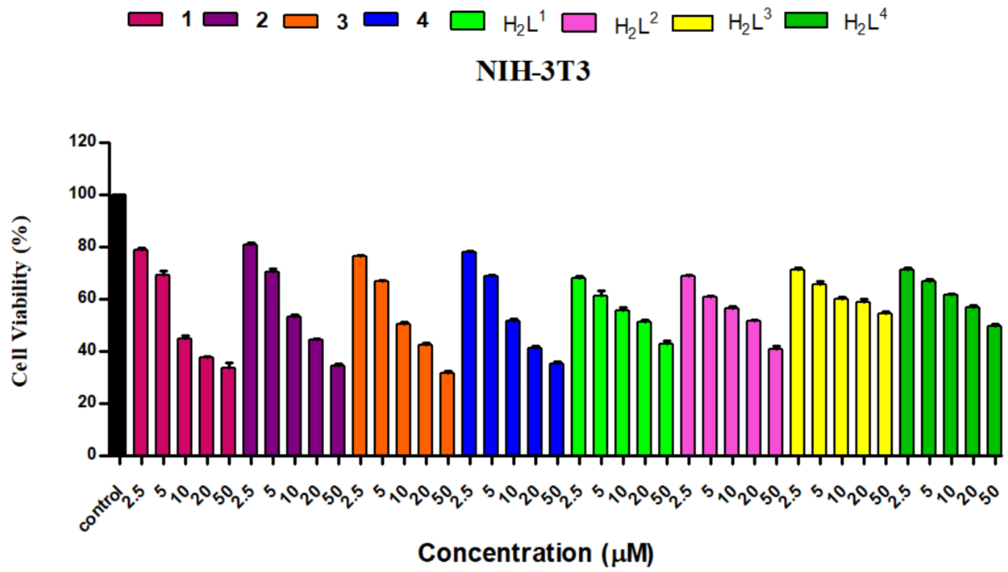

Figure S16. Cytotoxicity profiles of complexes **1**–**4** for NIH-3T3 cell lines after 48 h of incubation.

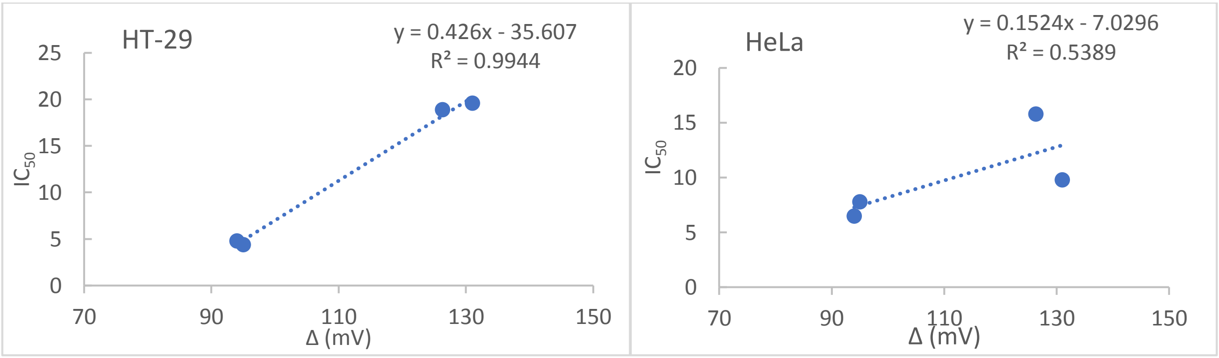

Figure S17. Correlations between the IC_{50} values and the gap (mV) between the oxidation and reduction potentials for complexes **1–4**.
